# Model-based dimensionality reduction for single-cell RNA-seq using generalized bilinear models

**DOI:** 10.1093/biostatistics/kxaf024

**Published:** 2025-08-09

**Authors:** Phillip B Nicol, Jeffrey W Miller

**Affiliations:** Department of Biostatistics, Harvard University, 677 Huntington Ave, Boston, MA, 02115, United States; Department of Biostatistics, Harvard University, 677 Huntington Ave, Boston, MA, 02115, United States

**Keywords:** dimension reduction, generalized linear model, single-cell RNA-sequencing

## Abstract

Dimensionality reduction is a critical step in the analysis of single-cell RNA-seq (scRNA-seq) data. The standard approach is to apply a transformation to the count matrix followed by principal components analysis (PCA). However, this approach can induce spurious heterogeneity and mask true biological variability. An alternative approach is to directly model the counts, but existing methods tend to be computationally intractable on large datasets and do not quantify uncertainty in the low-dimensional representation. To address these problems, we develop scGBM, a novel method for model-based dimensionality reduction of scRNA-seq data using a Poisson bilinear model. We introduce a fast estimation algorithm to fit the model using iteratively reweighted singular value decompositions, enabling the method to scale to datasets with millions of cells. Furthermore, scGBM quantifies the uncertainty in each cell’s latent position and leverages these uncertainties to assess the confidence associated with a given cell clustering. On real and simulated single-cell data, we find that scGBM produces low-dimensional embeddings that better capture relevant biological information while removing unwanted variation.

## 1. INTRODUCTION

Single-cell RNA sequencing (scRNA-seq) is a revolutionary technology that allows gene expression to be profiled at the level of individual cells (Saliba et al. 2014). This enables the identification of novel cell types that play critical roles in biological processes. However, the increased resolution provided by scRNA-seq comes at the cost of introducing several statistical and computational challenges. One major challenge is the large size of scRNA-seq datasets, which often contain millions of cells (Cao et al. 2019); thus, new methods must be computationally scalable (Lähnemann et al. 2020). Another challenge is that the extreme sparsity and discreteness of scRNA-seq count data make traditional statistical models based on normal distributions inappropriate (Vallejos et al. 2017). A third major challenge is that cell-level measurements are noisy and contain relatively little information compared to bulk sequencing, making it important to quantify the uncertainty in these measurements and propagate it to downstream analyses (Lähnemann et al. 2020).

Due to the large size of single-cell datasets, it is standard practice to use a dimensionality reduction technique such as principal components analysis (PCA) before clustering and other downstream analyses (Luecken and Theis 2019). Researchers typically apply a transformation to the count matrix before running PCA to avoid bias due to heteroscedasticity between different genes (Ahlmann-Eltze and Huber 2023). Unfortunately, existing research has demonstrated that commonly used transformations such as $ \log(1+x) $ can still lead to substantial bias in the subsequent PCA results (Lun 2018; Townes et al. 2019). This has motivated the current leading approach of transforming the counts using the Pearson residuals of a probabilistic model, as done by scTransform (Hafemeister and Satija 2019). However, Lause et al. (2021) have pointed out several biases in scTransform which, in turn, have been discussed by Choudhary and Satija (2022). Indeed, our work shows that scTransform and other Pearson residual-based methods can fail to capture signal in even simple simulations. The theoretical analysis of Warton (2018) demonstrated that no transformation can adequately stabilize variance for count distributions with a small mean, as is expected in scRNA-seq data.

Alternatively, using a probabilistic model of the count data matrix can avoid the artifactual biases of simple transformations. For example, GLM-PCA (Townes et al. 2019), ZINB-WAVE (Risso et al. 2018), and NewWave (Agostinis et al. 2022) use Poisson or negative binomial models for the counts—also allowing for zero inflation in the case of ZINB-WAVE—to estimate latent factors in log space. However, these methods suffer from slow runtime and convergence issues on large single-cell datasets with many cells (Townes et al. 2019; Lause et al. 2021). An advantage of a model-based approach to dimensionality reduction is that one can, in principle, quantify uncertainty in the low-dimensional representation of cells. Understanding the confidence in each cell’s latent position is useful for discerning whether the clusters represent biologically distinct groups as opposed to technical or computational artifacts. However, to the best of our knowledge, none of the existing methods use uncertainty in the low-dimensional embedding to provide uncertainty quantification in downstream analyses.

In this paper, we introduce scGBM, a novel approach to dimensionality reduction for scRNA-seq data. Starting from the same underlying model as in GLM-PCA (Townes et al. 2019), we provide three key innovations. First, we develop a new estimation algorithm that is faster than existing approaches and scales up to datasets with millions of cells. Second, we quantify uncertainty in the low-dimensional embedding, enabling calibrated inference in downstream analyses. Third, we use these uncertainties to define a *cluster cohesion index* (CCI) that provides a quantitative tool for assessing which clusters represent biologically distinct populations, rather than simply being artifacts of sampling variability. When analyzing numerous clusters, the CCIs reveal relationships that would be difficult to see with scatterplots. We evaluate the performance of scGBM on real and simulated data, finding that in many cases the current leading approaches are unable to capture true biological variability, while scGBM is successful in doing so.

The article is organized as follows. In Section 2, we demonstrate some of the limitations of current leading methods for scRNA-seq dimensionality reduction. In Section 3, we introduce our proposed methodology. Section 4 contains empirical results comparing the performance of our method versus leading approaches on simulated and real data. We conclude in Section 5 with a brief discussion. An scGBM R package is available for download (https://github.com/phillipnicol/scGBM).

## 2. MOTIVATION

In this section, we illustrate some limitations of the most commonly used approaches to dimensionality reduction for single-cell RNA-seq data. Let $ Y\in\mathbb{R}^{I\times J} $ be the matrix of unique molecular identifier (UMI) counts, where $ I $ is the number of genes and $ J $ is the number of cells. Define $ S_{j}=\sum_{i\,=\,1}^{I}Y_{ij} $ to be the sum of the counts for cell $ j $. Consider the following methods.

1. **Log+PCA**: This approach transforms the raw counts as
  (2.1) \begin{align*} Z_{ij}=\log\left(L\frac{Y_{ij}}{S_{j}}+1\right)\end{align*}
  where $ L=\mathrm{median}(S_{1},\ldots, S_{J}) $. Then, PCA is applied to the matrix $ Z\in\mathbb{R}^{I\times J} $. This is the default method used by *Scanpy* (Wolf et al. 2018; Heumos et al. 2023).
2. **Log+Scale+PCA**: The same transformation (2.1) is applied but with a fixed value of $ L=10^{4} $, followed by standardizing the rows of $ Z $ to have zero mean and unit variance. This is the default used by *Seurat* (Satija et al. 2015).
3. **SCT+PCA**: *scTransform* (Hafemeister and Satija 2019) fits the following model:
  (2.2) \begin{gather*} Y_{ij} \sim\mathrm{NegBinom}(\mu_{ij},\theta_{i})\nonumber\\\log(\mu_{ij})=\beta_{0i}+\beta_{1i}\log(S_{j}),\tag{2.2}\end{gather*}
  where $ \mathrm{NegBinom}(\mu , \theta) $ is the negative binomial distribution with mean $ \mu $ and dispersion $ 1/\theta $. The resulting estimates of $ \hat{\mu}_{ij} $ and $ \hat{\theta}_{i} $ are used to compute the Pearson residuals, defined as
  (2.3) \begin{align*} Z_{ij}:=\frac{Y_{ij}-\hat{\mu}_{ij}}{\sqrt{\hat{\mu}_{ij}+\hat{\mu}_{ij}^{2}/\hat{\theta}_{i}}}.\end{align*}
  Then, PCA is applied to the matrix $ Z\in\mathbb{R}^{I\times J} $.
4. **APR+PCA**: Analytic Pearson residuals (Lause et al. 2021) are computed using (2.3) but with a fixed value of $ \hat{\theta}_{i}=100 $ and a closed-form approximation to $ \hat{\mu}_{ij} $.

### 2.1. Single marker genes simulation

**Fig. 1.**
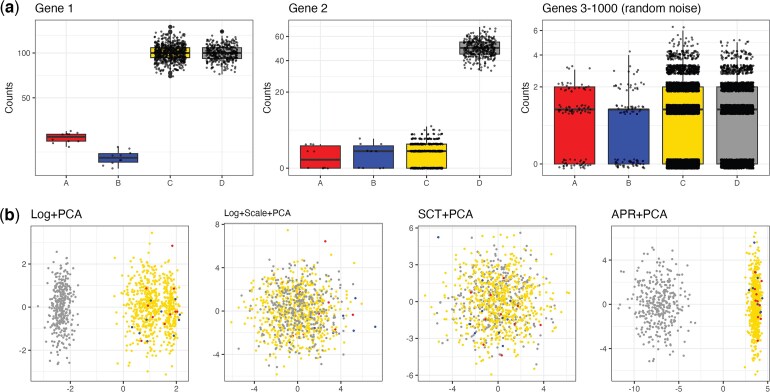
Single marker gene simulation. a) Boxplots of simulated for 1000 cells from 4 different cell types. Gene 1 is low in cell types A and B. Gene 2 is high in cell type D. The remaining genes are $ \mathrm{Poisson}(1) $ random noise. b) The scores obtained from applying PCA to the four approaches based on transforming the count matrix prior to PCA.

As mentioned above, previous works have discussed potential shortcomings of applying a normalization prior to PCA (Lun 2018; Townes et al. 2019). Here, we construct a simple simulation where these approaches fail to capture biological signal. We consider a simulation with two rare cell types ($ A $ and $ B $) and two common cell types ($ C $ and $ D $). The four cell types ($ A $, $ B $, $ C $, and $ D $) are distinguished by their mean expression for gene 1 (moderate, low, high, and high, respectively) and gene 2 (low, low, low, and high, respectively); see Fig.  1a and Section S3. The remaining 998 null genes are drawn i.i.d. from a $ \mathrm{Poisson}(1) $ distribution. We evaluate the ability of each method to separate the cell types via the first two PCs. In Fig.  1b, we see that Log+Scale+PCA and SCT+PCA fail to separate any of the cell types. While Log+PCA and APR+PCA are able to separate the common cell types ($ C $ and $ D $), they are not able to separate the rare cell types ($ A $ and $ B $) from cell type $ C $ or from one another. This occurs because the variances of genes 1 and 2 (after transformation) are significantly smaller than the leading eigenvalues from PCA, indicating that the normalization procedure downweights the signal and upweights the noise due to null genes (Fig. S1). Uniform Manifold Approximation and Projection (UMAP) (McInnes et al. 2018) does not help separate out the rare cell types; see Fig. S2 for UMAP applied to the top 10 principal components from each method.

To investigate further, we conduct a sensitivity analysis in which we vary the settings of this simulation; see Section S3 for details. We find that as the size of the rare cell type increases beyond 25 cells, scGBM, APR+PCA, and (for low values for the mean expression of the rare cell type) Log+PCA are all able to successfully separate the rare cell type, while SCT+PCA still struggles; see Fig. S3. Although this example is intended to be illustrative rather than biologically realistic, we show on real data in Section 4.2 that the presence of rare cell types can negatively influence downstream results more generally when using the PCA-based methods.

## 3. METHODS

In this section, we introduce our proposed model (Section 3.1), our iteratively reweighted singular value decomposition (Section 3.2), our projection technique for scaling to large matrices (Section 3.3), and our uncertainty quantification approach (Section 3.4).

### 3.1. scGBM fits a Poisson bilinear model to the count matrix

Our proposed method, referred to as scGBM, handles normalization, transformation, and latent factorization in a unified way using a Poisson bilinear model. Specifically, we model the entries of the UMI count matrix $ Y\in\mathbb{R}^{I\times J} $ (with genes on rows and cells on columns) as


(3.4) \begin{gather*} Y_{ij}\sim\mathrm{Poisson}(\mu_{ij})\nonumber\\\log(\mu_{ij}) = \alpha_{i}+\beta_{j}+\sum_{m=1}^{M}\sigma_{m}u_{im}v_{jm}\tag{3.4}\end{gather*}

for $ i\,=\,1 , \ldots, I $ and $ j\,=\,1 , \ldots, J $, where $ \alpha_{i} $ is a gene-specific intercept, $ \beta_{j} $ is a cell-specific intercept, $ U:=[u_{im}]\in\mathbb{R}^{I\times M} $ is a matrix of latent factor loadings or weights, $ V:=[v_{jm}]\in\mathbb{R}^{J\times M} $ is a matrix of latent factor scores, and $ \Sigma:=\mathrm{diag}(\sigma_{1},\ldots , \sigma_{M})\in\mathbb{R}^{M\times M} $ is a diagonal matrix of scaling factors (or singular values) $ \sigma_{m} > 0 $. We use a prior $ \sigma_{m}\sim\mathrm{Exponential}(\tau) $ to improve stability. For intuition, note that when the link function is the identity instead of the logarithm, and the outcome distribution is Gaussian instead of Poisson, then the estimates of $ \sigma $, $ U $, and $ V $ can simply be obtained using PCA (Miller and Carter 2020). Thus, this model can roughly be thought of as analogous to PCA inside the link function of a generalized linear model (GLM). Identifiability constraints and more background on the model are provided in Section S1.1. We also note that when $ M\,=\,0 $ (no latent factors) and $ \beta_{j}=\log(S_{j}) $, scGBM is equivalent to the starting model for analytic Pearson residuals (Lause et al. 2021). Our proposed estimation algorithm leverages this connection for initializing the parameter estimates; see Proposition 1 in the Supplementary Materials.

Following Choulakian (1996), we refer to the model in (3.4) as a *generalized bilinear model* (GBM), but other names such as GAMMI (Van Eeuwijk 1995) or eSVD (Lin et al. 2021) have been used in the statistical literature. We refer to Miller and Carter (2020) for an extensive discussion of the literature on GBMs. In the single-cell literature, (3.4) is typically referred to as GLM-PCA (Townes et al. 2019). Although scGBM is based on the same model as GLM-PCA, there are some key differences between the two methods. First, scGBM is able to estimate cell-specific intercepts $ \beta_{j} $, whereas GLM-PCA treats the $ \beta_{j} $’s as fixed offsets. Second, GLM-PCA does not enforce identifiability constraints in the intercepts or the factors. Finally, GLM-PCA does not quantify uncertainty in the parameter estimates.

In contrast to many bulk RNA-seq models, the scGBM model assumes a Poisson outcome and thus does not allow for overdispersion. In fact, there is increasing evidence that the technical sampling distribution of UMI counts is close to Poisson and that any additional dispersion is due to biological variability such as heterogeneous cell types or cell states (Lause et al. 2021; Sarkar and Stephens 2021). Since the goal of scGBM is to remove technical variability while preserving all biological variability for downstream analyses, it is natural to use a Poisson outcome distribution.

### 3.2. Fast and stable estimation using iteratively reweighted singular value decomposition

Existing methods for fitting GBMs are not scalable to the large datasets encountered in scRNA-seq and are known to suffer from convergence issues. To address these limitations, we propose a new approach to fitting the Poisson GBM that combines iteratively reweighted least squares (IRLS) and singular value decomposition (SVD). This pairing is natural since IRLS is the standard way to fit GLMs and SVD is the standard way to perform PCA.

Define $ X\,=\,U\Sigma V^{T} $, and suppose we have current estimates $ \hat{\alpha} $, $ \hat{\beta} $, and $ \hat{X} $. By a second-order Taylor approximation at these estimates (see Section S1.2 for details), maximizing the log-posterior of the scGBM model in (3.4) is approximately equivalent to solving the weighted low-rank problem


(3.5) \begin{align*}\min\limits_{X\,:\,\mathrm{rank}(X)=M}\sum\limits_{i, j}W_{ij}(Z_{ij}-X_{ij})^{2}+\tau\|X\|_{*}\end{align*}

where $ W_{ij}=\hat{\mu}_{ij}/\hat{\mu}_{*} $, $ \hat{\mu}_{*}=\max_{ij}\hat{\mu}_{ij} $, $ Z_{ij}=\hat{X}_{ij}+(Y_{ij}-\hat{\mu}_{ij})/\hat{\mu}_{ij} $, $ \hat{\mu}_{ij}=\exp(\hat{\alpha}_{i}+\hat{\beta}_{j}+\hat{X}_{ij}) $, and $ \|\cdot\|_{*} $ denotes the nuclear norm, that is, the sum of the singular values. Tuzhilina and Hastie (2021) introduced a simple and efficient method for solving this type of weighted low-rank problem (with fixed $ W $ and $ Z $) by iteratively applying the equation


(3.6) \begin{align*}\hat{X}^{\mathrm{(new)}}=\mathrm{SVD}_{M , \tau}\big(\hat{X}+W\circ(Z-\hat{X})\big)\end{align*}

where $ \mathrm{SVD}_{M , \tau}(\cdot) $ returns the rank $ M $ truncated SVD of a matrix with the leading $ M $ singular values soft-thresholded by $ \tau $, and $ \circ $ is the Hadamard product. In turn, when $ \hat{X} $ is held fixed, the maximum likelihood estimates of $ \hat{\alpha} $ and $ \hat{\beta} $ have closed-form solutions.

Thus, we alternate between updating $ \hat{X} $ by applying (3.6) with $ \hat{\alpha} $ and $ \hat{\beta} $ fixed, and updating $ \hat{\alpha} $ and $ \hat{\beta} $ to their maximum likelihood estimates with $ \hat{X} $ fixed. The truncated SVD automatically factors $ \hat{X} $ as $ \hat{U}\hat{\Sigma}\hat{V}^{T} $, yielding estimates of the loadings, scores, and singular values. See Section S1.3 for a step-by-step description of the proposed algorithm, which we call *iteratively reweighted singular value decomposition* (IRSVD).

There are several advantages of the IRSVD algorithm. First, it is asymptotically faster than Fisher scoring, the technique used by Miller and Carter (2020) and Townes et al. (2019); see Section S1.4 for the computational complexity. Further, one can leverage special properties of Poisson GLMs to obtain vectorized updates for the intercepts—that is, the entries of $ \alpha $ and $ \beta $ can be updated simultaneously—which greatly reduces the runtime in practice. Finally, the identifiability constraints such as orthogonality are preserved at every iteration of the algorithm.

### 3.3. Scaling up by fitting on a subset of samples and projecting

Even with the improvement due to IRSVD, the computation time scales linearly with the number of samples, making it burdensome on datasets with millions of cells. Thus, to further improve scalability, we propose using a combination of subsampling and projection. Specifically, we first randomly select a subset of cells and run the algorithm in Section S1.3 to estimate the parameters using only the data from these cells. Then, holding $ U $ and $ \alpha $ fixed at these estimated values, column $ j $ of the Poisson bilinear model in (3.4) is simply a GLM with covariate matrix $ U $ and coefficients $ \beta_{j},\sigma_{1}v_{j1},\ldots , \sigma_{M}v_{jM} $, which can be fit using standard software. We refer to this as the “projection method.”

We refer to this subsampling-projection version of the algorithm as “scGBM-proj,” in contrast to running the algorithm on the full dataset, which we refer to as “scGBM-full.” The observation that fixing $ U $ or $ V $ yields a standard GLM has been used in previous RNA-seq techniques (Leek and Storey 2007; Sun et al. 2012; Risso et al. 2014). Importantly, the GLMs can be fit in parallel since each column is processed independently. This also reduces memory requirements since the count matrix $ Y $ can remain stored in sparse form and only needs to be loaded into memory one column at a time, making it suitable for on-disk processing tools like *DelayedArray* (Pagès 2020).

### 3.4. Quantifying uncertainty in the latent factors

Since scGBM performs dimensionality reduction using a statistical model, uncertainty in the low-dimensional representation can be quantified using the classical method of inverting the Fisher information matrix. Miller and Carter (2020) use this approach (see Section S6.1), accounting for the joint uncertainty in $ U $ and $ V $ and also their identifiability constraints. However, this is computationally expensive on single-cell datasets.

Thus, to quantify uncertainty in $ V $, we use a rough approximation by inverting the diagonal blocks of the Fisher information for $ V $, that is, the submatrices $ F_{1},\ldots, F_{J}\in\mathbb{R}^{M\times M} $ in which entry $ (m, m^{\prime}) $ of $ F_{j} $ is


(3.7) \begin{align*} F_{j, m, m^{\prime}}=-E\bigg(\frac{\partial^{2}\ell}{\partial v_{jm}\partial v _{jm^{\prime}}}\bigg)\end{align*}

for $ j\,=\,1 , \ldots, J $ and $ m, m^{\prime}=1 , \ldots, M $, where $ \ell $ is the log-likelihood. For our model (3.4),


(3.8) \begin{align*}\frac{\partial^{2}\ell}{\partial v_{jm}\partial v_{jm^{\prime}}}=-\sum\limits_{i=1}^{I}\sigma_{m}u_{im}\mu_{ij}\sigma_{m^{\prime}}u_{im^{\prime}}\end{align*}

by differentiating the log-likelihood $ \ell $. Therefore, in matrix form,


(3.9) \begin{align*} F_{j}=(U\Sigma)^{T}\tilde{W}_{j}(U\Sigma)\end{align*}

where $ \tilde{W}_{j}=\mathrm{diag}(\mu_{1j},\ldots , \mu_{Ij})\in\mathbb{R}^{I\times I} $. By plugging in parameter estimates and taking the square root of the diagonal entries of $ F_{j}^{-1} $, we obtain approximate standard errors for the estimates of $ v_{j1},\ldots, v_{jM} $. This approximation will tend to underestimate the uncertainty in $ V $ since it treats the other parameters $ \alpha $, $ \beta $, $ U $, and $ \Sigma $ as fixed. Nonetheless, on simulated data, we find that it yields confidence intervals with coverage only slightly below the target coverage, for large $ I $ and $ J $ (Fig. S4). Standard errors for $ U $ can be approximated in a complementary way.

## 4. RESULTS

### 4.1. Comparing to GLM-PCA

In this section, we compare the performance of our estimation algorithm to three algorithms provided by the GLM-PCA R package as implemented by Townes and Street (2020): Fisher scoring, Avagrad (Savarese et al. 2021), and stochastic gradient descent (SGD). We generated simulated datasets with $ I\,=\,1000 $, $ J\in\{2000,10^{5}\} $, and $ M\,=\,10 $ by sampling $ U $ and $ V $ uniformly from the Stiefel manifold, setting $ \Sigma=\mathrm{diag}(\sigma_{1},\ldots , \sigma_{M}) $ where $ \sigma_{m}=(\kappa m/M)(\sqrt{I}+\sqrt{J}) $, generating intercepts $ \alpha_{i} $ and $ \beta_{j} $ independently from $ \mathcal{N}(0,1) $, and generating $ Y $ according (3.4). On each dataset, we ran the estimation algorithms to produce parameter estimates $ \hat{U} $, $ \hat{V} $, and $ \hat{\Sigma}=\mathrm{diag}(\hat{\sigma}_{1},\ldots , \hat{\sigma}_{M}) $.

**Fig. 2.**
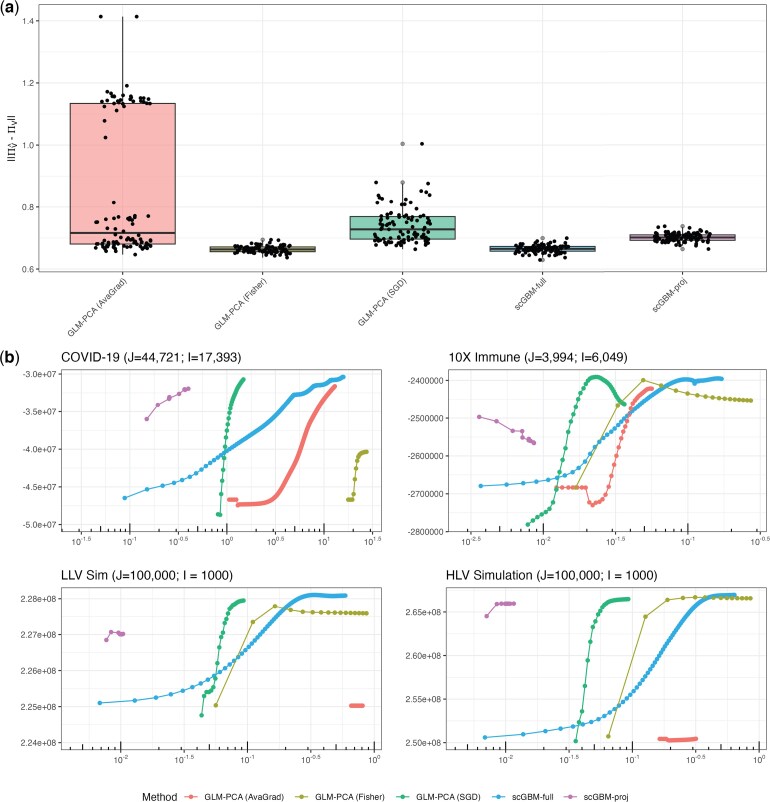
Comparison to GLM-PCA. a) For 100 simulated datasets with $ J\,=\,10{,}000 $ and $ I\,=\,1{,}000 $, $ \|\Pi_{\hat{V}}-\Pi_{V}\|_{F} $ is plotted where $ \Pi_{V}:=V(V^{\top}V)^{-1}V^{\top} $. b) Out-of-sample log-likelihood versus wall clock time on two real datasets and two simulated datasets (HLV with $ \kappa\,=\,5 $ and LLV with $ \kappa\,=\,2 $). For the two real datasets, the out-of-sample dataset is obtained by count splitting (Neufeld et al. 2023). For the simulated data, the out-of-sample log-likelihood is computed as $ \sum_{i, j}(\mu_{ij}\log(\hat{\mu}_{ij})-\hat{\mu}_{ij}) $ since the ground truth is known in this case.

To assess accuracy, we simulated 100 datasets as above with $ J\,=\,10{,}000 $ and $ \kappa\,=\,2 $ and computed $ \|\Pi_{\hat{V}}-\Pi_{V}\|_{F} $ where $ \Pi_{V}=V(V^{\top}V)^{-1}V^{\top} $ is the projection onto the column space of $ V $. This metric was used because it does not depend on a particular choice of basis and instead compares the subspaces of $ \mathbb{R}^{J} $ spanned by columns of $ V $ and $ \hat{V} $. We found that GLM-PCA (Avagrad) and GLM-PCA (SGD) performed significantly worse than both scGBM methods (Fig.  2a). Atlhough GLM-PCA (Fisher) performed comparably to scGBM-full by this metric, our subsequent results show that it is computationally prohibitive on larger datasets.

To measure the performance on real data, we use count splitting to obtain train and test data sets (Neufeld et al. 2023). Specifically, defining $ Y^{*}_{ij}\sim\mathrm{Binomial}(Y_{ij},1/2) $, $ Y^{**}_{ij}:=Y_{ij}-Y^{*}_{ij} $, and assuming the counts are Poisson distributed, it follows that $ Y^{*}_{ij} $ and $ Y^{**}_{ij} $ are independent and identically distributed for any given $ i, j $. Thus, we fit the model to $ Y^{**} $ and define the out-of-sample log-likelihood (with additive constants removed) as $ \sum_{i, j}(Y_{ij}^{*}\log(\hat{\mu}_{ij})-\hat{\mu}_{ij}) $. Figure  2b shows the out-of-sample log-likelihood versus the wall clock time, that is, the elapsed time since the start of the algorithm. We performed this analysis on 10X immune cells ($ J\,=\,3{,}994 $, Zheng et al. 2017) and a COVID-19 Atlas ($ J\,=\,44{,}721 $, Wilk et al. 2020). On both datasets, scGBM-full converges at a higher out-of-sample log-likelihood than the three GLM-PCA methods (Fig.  2b). Note that on the 10X immune cell data, GLM-PCA (SGD) and GLM-PCA (Fisher) attain a maximum and begin decreasing before the algorithms converge, suggesting that they are overfitting the training data. The same overfitting also appears to occur for scGBM-proj on this dataset and may be due to insufficient subsample size. On small datasets such as this, however, the speed increase of scGBM-proj is not needed. We only recommend using scGBM-proj in situations where it is computationally infeasible to apply scGBM-full. The embeddings for each method on the two datasets are shown in Figs S6 and S8. In particular, we observe that PCA-based methods tend to “stretch” certain cell types more than scGBM/GLM-PCA. After applying UMAP, however, embeddings appeared qualitatively similar; see Figs S7 and S9. Next, we performed the same analysis on two simulated datasets with $ J\,=\,100{,}000 $ cells; one with “low latent variability” (LLV, $ \kappa\,=\,2) $ and one with “high latent variability” (HLV, $ \kappa\,=\,5 $). On the simulated data, there is no need to split the counts since the ground truth is known, so we fit using the full counts and assess performance via $ \sum_{i, j}(\mu_{ij}\log(\hat{\mu}_{ij})-\hat{\mu}_{ij}) $. Again, we found that scGBM-full performed the best, with the greatest difference occurring in the LLV simulation (Fig.  2b).

We applied the same analyses to test the performance of our projection method (scGBM-proj). On the simulated data, scGBM-proj (using 1000 subsamples) achieved only a slightly lower accuracy than scGBM-full (Fig.  2a). On the real data, scGBM-proj converged to a reasonable solution 10-100x faster than competing methods (Fig.  2b). We also compared the magnitude of the correlation between each factor of scGBM-proj and scGBM-full, finding strong agreement between the leading factors estimated by both approaches (Figs S11 and S12). We also note that due to the nature of the algorithms, scGBM-proj with 100% subset size is not equivalent to scGBM-full, although Fig. S13 shows that the most important leading factors are nearly identical between the two methods.

The projection approach allows scGBM to be applied to large scRNA-seq datasets with millions of cells. For example, on 10X mouse brain data (Lun and Morgan 2020) with $ J\,=\,1{,}308{,}421 $ cells, scGBM-proj finished in 57 min whereas GLM-PCA and NewWave (Agostinis et al. 2022) were orders of magnitude slower.

### 4.2. Comparing to transformation prior to PCA

**Fig. 3.**
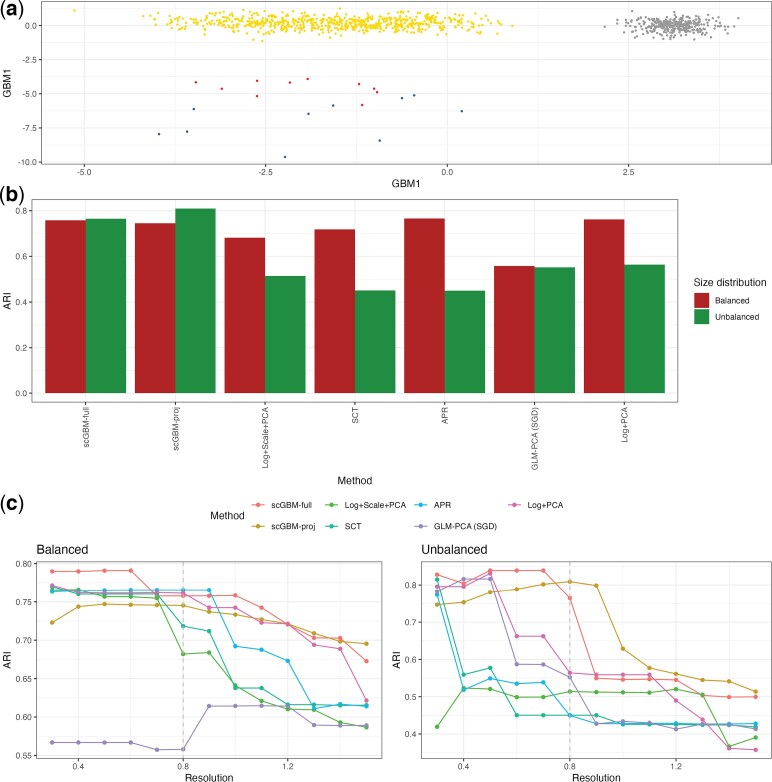
Performance of scGBM on real and simulated data. a) The top two GBM factors on the data from the single marker genes simulation. b) For the “Balanced” setting, each method was applied to the 10X immune cell data and clusters were obtained using the Louvain algorithm with the resolution of 0.8. Then the ARI was computed using the ground truth labels. For the “Unbalanced” setting, we repeated the same analysis but first downsampled the 10X immune cell data such that one cell type was dominant and the others were rare. c) ARI plotted across different choices of the resolution parameter of the clustering algorithm for both the balanced and unbalanced dataset. The dashed vertical line marks the Seurat default resolution of 0.8. We use GLM-PCA (SGD) here since it was the best performing variant of GLM-PCA in our simulations in Fig. 2.

We return to the single marker gene simulation from Fig. 1 and observe that, unlike PCA-based methods, scGBM is able to clearly separate the four cell types (Fig.  3a). In particular, the subtle difference between cell types $ A $ and $ B $ is also reflected in the second scGBM score. By directly modeling the counts, scGBM is able to detect variation that is lost when the data are normalized prior to dimension reduction.

To evaluate the practical benefit of using scGBM, we assessed clustering performance using the 10X immune cell labels (Zheng et al. 2017) as ground truth. For each method, we produced a 20-dimensional embedding and applied the Louvain clustering algorithm using a resolution of 0.8 (Blondel et al. 2008), which is the default in Seurat. We then assessed performance in terms of the adjusted Rand index (ARI) (Rand 1971) between the inferred clusters and the ground truth. For this analysis, scGBM-full, scGBM-proj, and APR+PCA performed the best, while GLM-PCA struggled (Fig.  3b). To evaluate the effect of class size imbalance, we then considered a data set produced by subsampling the 10X immune cell data to have one dominant cell type and several uncommon cell types (see Section S3 for details). On this unbalanced cell type size data, scGBM-full and scGBM-proj significantly outperformed the other methods (Fig.  3b). We tested the sensitivity of these results to the choice of resolution, finding that scGBM-full and scGBM-proj outperformed competing methods at a majority of resolutions between 0.3 and 1.5 (Fig.  3c).

To better understand reason that scGBM performed better in the presence of class imbalance, we produced UMAP visualizations for each method in Fig. S10. These plots revealed that each of the PCA-based methods divide the monocyte cluster into 3 subclusters. To test if these subclusters constitute biologically meaningful subpopulations, we again used the method of count splitting to create two i.i.d. datasets $ Y_{1} $ and $ Y_{2} $. If the subclusters identified in Fig. S10 were meaningful, then the monocyte clusterings of $ Y_{1} $ and $ Y_{2} $ should be similar. That is, monocytes that cluster together in the first dataset should also cluster together in the second dataset. We compute the fraction of pairs of cells that were clustered together in $ Y_{1} $ and $ Y_{2} $. Table S2 shows the median of this fraction across 5 count splitting iterations. In particular, the PCA-based methods produce inconsistent clusterings of the monocytes on count-split data, suggesting that the clusters identified in Fig. S10 may be spurious.

We also considered downsampling the COVID-19 data to create class imbalance, again finding that scGBM outperformed PCA-based methods (Table S3).

### 4.3. Uncertainty quantification and the cluster cohesion index

Clustering typically occurs downstream of dimensionality reduction, as a *post hoc* analysis step (Kiselev et al. 2019). Uncertainty in the estimated low-dimensional representation is likely to influence clustering results and other downstream analyses, but the effect of this uncertainty has not been thoroughly investigated in previous work. For example, one might wonder whether different clusters would be identified if the same set of cells was re-sequenced and the same analysis was performed on the resulting new count matrix.

To illustrate, we simulated a count matrix consisting of random Poisson noise, $ Y_{ij}\sim\mathrm{Poisson}(1) $ i.i.d. for $ i\,=\,1 , \ldots, I $ and $ j\,=\,1 , \ldots, J $ with $ I\,=\,1000 $ and $ J\,=\,5000 $, and we used scGBM to obtain estimated scores $ \hat{V} $. Then we applied the Louvain algorithm (Blondel et al. 2008) to $ \hat{V} $ to identify clusters of cells, since this is the default clustering algorithm used in Seurat (Satija et al. 2015) (FindClusters() with default resolution equal to 0.8). The Louvain algorithm identified 9 clusters, even though the cells were simulated to be homogeneous. The fact that standard single-cell clustering algorithms produce too many clusters on null data such as this has also been noted in other studies (Morelli et al. 2021; Grabski et al. 2022).

**Fig. 4.**
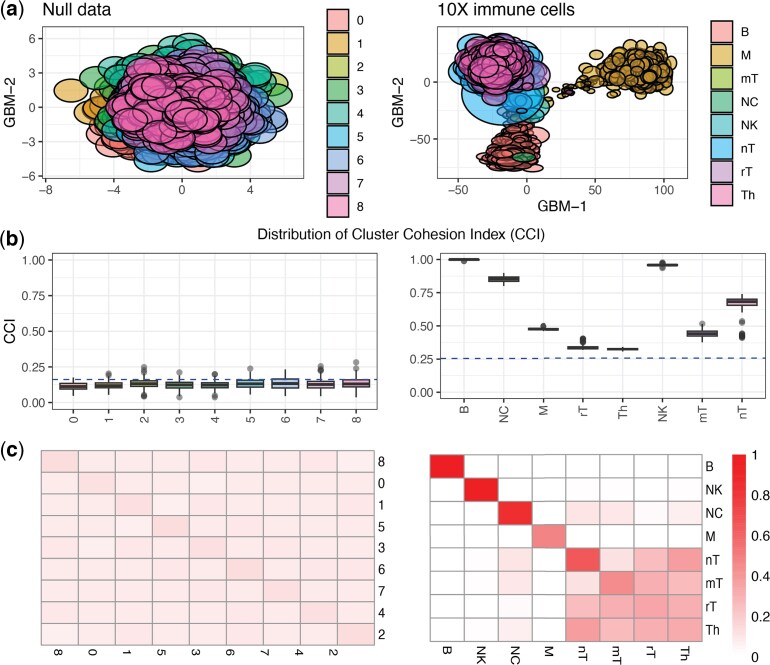
Cluster cohesion results for two datasets: random Poisson noise and the 10X immune cells. In the 10X immune data, B = “B cells,” M =“Monocytes,” mT = “Memory T,” NC = “Naive cytotoxic,” NK = “Natural Killer,” nT = “Naive T,” rT = “Regulatory T,” and Th = “T helper.” a) The first two columns of $ \hat{V}\hat{\Sigma} $. The uncertainty in each point is visualized by drawing an ellipse with axis lengths equal to the estimated standard errors. b) The distribution of the CCI fractions $ f_{k, k} $; see (S1.35). c) Heatmaps of inter-CCIs for the random Poisson noise data and the 10X immune cells dataset. The inter-CCIs enable one to visualize the uncertainty in the distinctions between different clusters.

With scGBM, we can use the uncertainty in $ \hat{V} $ to quantify the uncertainty in these clusters. First, a simple visualization of the uncertainty in $ \hat{V} $ can be obtained by drawing an ellipse around each point, with the dimensions of the ellipse equal to the estimated standard errors; see Fig.  4a (left). The considerable overlap among these ellipses suggests that there are no clearly separated clusters. Meanwhile, when there are true clusters, the ellipses for cells from different clusters tend not to overlap as much. For example, on the 10X immune cell data, there is relatively little overlap between the uncertainty ellipses for the distinct cell populations (T cells, B cells, monocytes); see Fig.  4a (right). However, the qualitative visualizations in Fig.  4a may be difficult to interpret or use, especially when there are many clusters.

To provide a quantitative measure of the uncertainty in each cluster, we introduce the *cluster cohesion index* (CCI). Given estimates $ \hat{v}_{jm} $ and corresponding standard errors $ \mathrm{se}(\hat{v}_{jm}) $, we randomly generate perturbed values $ \tilde{v}_{jm}\sim\mathcal{N}(\hat{v}_{jm},\mathrm{se}(\hat{v}_{jm})^{2}) $ independently, perform clustering on the rows of $ \tilde{V}=[\tilde{v}_{jm}] $, and compute the fraction of pairs of cells that are still in the same cluster. Then, for each original cluster, the CCI is defined as the mean of this fraction over many repetitions; see Section S1.5 for details. The CCI takes a value between 0 and 1, with 1 indicating that all pairs of cells are always still in the same cluster after resampling.

Figure  4b shows the CCIs for the random Poisson noise dataset and the 10X immune cell dataset from Fig.  4a. As expected, the CCIs for the random noise dataset are all low, reflecting the fact that any identified clusters are artifacts of sampling variability. Meanwhile, on the 10X immune cell data, the CCIs for the true cell types are all higher. We can define a significance threshold for CCIs by computing the values that would have been generated under the null model of $ V\,=\,0 $; see Section S1.5 for details. As one would hope, none of the random Poisson noise CCIs reach the significance threshold, while all of the 10X immune cell CCIs are past the threshold; see dashed blue lines in Fig.  4b.

To quantify the overlap between clusters in a way that accounts for model-based uncertainty, we define the *inter-cluster cohesion index* (inter-CCI) as follows: out of all pairs of points $ j $ and $ j^{\prime} $ that were originally in clusters $ k $ and $ k^{\prime} $, respectively, the inter-CCI is the mean of the fraction that are in the same cluster after resampling; see Section S1.5 for details. Note that the inter-CCI between a cluster and itself coincides with the CCI of that cluster.

**Fig. 5.**
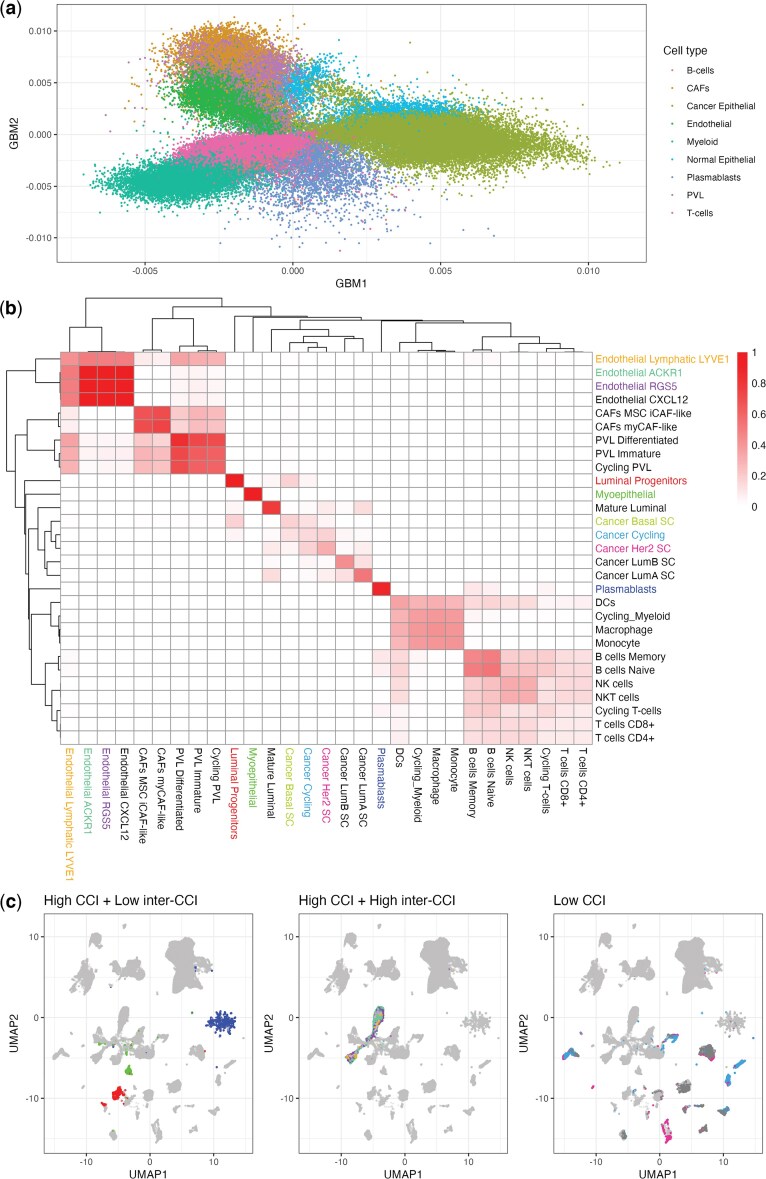
a) We applied scGBM to the Wu et al. (2021) dataset consisting of 100,064 breast cancer cells and plotted the first two scGBM scores (columns of $ \hat{V} $) colored by major cell type. b) The heatmap of inter-cluster cohesion indices (inter-CCIs) for the minor cell types. c) We plotted the scGBM+UMAP embedding with each color indicating 1 of 3 cell types with high CCI + low inter-CCI (Luminal progenitors, Myoepithelial, Plasmablasts), high CCI + high inter-CCI (Endothelial LYVE1, ACKR1, RGS5), or low CCI (Cancer Basal SC, Cycling, HER2 SC). The colors in panel b) correspond to the colors in panel c).

The inter-CCIs can be visualized using a heatmap (Fig.  4c). For the random Poisson noise data, all of the inter-CCIs are of similar magnitude to the CCIs themselves, indicating low confidence in all of the clusters, as expected. On the 10X immune cell data, the inter-CCIs for the T-cell subpopulations (nT, mT, rT, Th) are also relatively high, indicating lower confidence in the distinction between these groups. Meanwhile, the remaining subpopulations (B, NK, NC, and M) have high CCIs and low inter-CCIs, indicating high confidence in these clusters. The inter-CCIs allow one to visualize and quantify cluster relationships that would be difficult to discern using scatterplots alone.

To further demonstrate the utility of inter-CCIs, we consider a dataset of $ 100{,}064 $ cells from the tumor microenvironment of 26 breast cancer patients (Wu et al. 2021); see Fig.  5a. Since Wu et al. (2021) used the standard Seurat pipeline (Satija et al. 2015) to cluster cells and used Garnett (Pliner et al. 2019) for annotation, we computed the inter-CCIs for this Seurat-based clustering; see heatmap in Fig.  5b. The blocks along the diagonal indicate groups of cell types with substantial overlap when accounting for model-based uncertainty. These blocks appear to correspond to biologically relevant classes of cells such as lymphoid (T cells, B cells, NK cells), myleoid (monocytes and DCs), and endothelial cells.

The inter-CCI heatmap enables one to see the relationships among all 29 minor cell types, which would be difficult or impossible to see with a scatterplot. We then applied UMAP to the GBM scores, highlighting cell types that had either high CCI and low inter-CCI, high CCI and high inter-CCI, or low CCI (Fig. 5c). Each cell type with high CCI and low inter-CCI tends to be tightly clustered and not overlapping with other cell types. Thus, the quantitative measure of separation provided by the CCI could be used in conjunction with UMAP to validate the authenticity of the clusters. In contrast, the cell types with high CCI and high inter-CCI tend to be tightly clustered and strongly overlapping with one another. Thus, these cell types could potentially be combined for downstream analyses. Finally, each cell type with low CCI tends to be dispersed throughout the UMAP plot, indicating that it might consist of heterogeneous subtypes or might be poorly defined. We also applied UMAP to the embeddings produced by Log+Scale+PCA, Log+PCA, and APR+PCA on the same data set (Fig. S15). While these plots are qualitatively similar to scGBM+UMAP, the CCI cannot be used with these approaches because they do not provide uncertainty quantification.

## 5. DISCUSSION

Standard practice is to visualize single-cell data by applying UMAP or t-SNE (Van der Maaten and Hinton 2008) to the PCA scores (Becht et al. 2018; Kobak and Berens 2019). Our results in Fig. 1 and Figs S2 and S16 show that biases in PCA can propagate to the UMAP plots. Since we have found scGBM to largely overcome these limitations, we recommend using scGBM embeddings rather than PCA as input to UMAP, if one uses UMAP. However, it is known that UMAP can introduce distortions in two-dimensional visualizations (Wang et al. 2023), and contradictory conclusions have been reached on the utility of UMAP (Chari and Pachter 2023; Lause et al. 2024). Ultimately, the choice of whether or not to use UMAP alongside scGBM is up to the user.

In future work, there are several interesting directions for improving upon our current approach. Although our scGBM algorithm is faster than GLM-PCA, it is still much slower than PCA—especially because commonly used transformations preserve sparsity, which allows for the use of more efficient SVD algorithms (Baglama and Reichel 2005). This limitation could potentially be addressed by modifying our IRSVD algorithm to avoid applying the SVD to a dense matrix. A second limitation of scGBM is that, unlike PCA, the latent factors depend on the choice of $ M $. Although there are heuristics for choosing the number of latent factors $ M $, developing principled techniques for choosing $ M $ remains an area for future work. In this paper, we mainly considered quantifying uncertainty in the scores $ V $, but it would be also be interesting to develop additional approaches that use the uncertainty in the gene loadings $ U $. For example, one could propagate uncertainty into gene set enrichment analysis (Subramanian et al. 2005). A final area for future research would be to induce sparsity in the weights $ U $, to improve biological interpretability. Sparsity in $ U $ would mean that only a few genes have non-zero weights, making it easier to connect the factors to known biology, for instance, in terms of gene sets and pathways. To this end, it would be interesting to explore whether ideas from sparse PCA (Zou et al. 2006) can be extended to bilinear models.

## Supplementary Material

kxaf024_Supplementary_Data
