# Supplementary material for: Model-based dimensionality reduction for single-cell RNA-seq using generalized bilinear models
Source: Biostatistics. 2025 Aug 9;26(1):kxaf024. doi: 10.1093/biostatistics/kxaf024 (PMC12342792; doi:10.1093/biostatistics/kxaf024)
Supplement: kxaf024_Supplementary_Data [file kxaf024_supplementary_data.pdf]

Supplementary material for “Model-based dimensionality reduction for single-cell RNA-seq using generalized bilinear models”

S1. Extended Methods

S1.1 *Generalized bilinear model*

The scGBM method employs the Poisson bilinear model in Equation 3.4 for the matrix $Y \in \mathbb{R}^{I \times J}$ of UMI counts (I genes and J cells). If we define $\mu := [\mu_{ij}] \in \mathbb{R}^{I \times J}$ then Equation 3.4 can be rewritten in matrix form as

$$Y \sim \text{Poisson}(\mu)$$

$$\log(\mu) = \alpha \mathbf{1}_J^T + \mathbf{1}_I \beta^T + U \Sigma V^T$$

where Poisson and log are applied entry-wise, $\mathbf{1}_K = (1, \ldots, 1)^T \in \mathbb{R}^K$ is a vector of ones, and $\Sigma = \text{diag}(\sigma_1, \ldots, \sigma_M) \in \mathbb{R}^{M \times M}$. Theorem 5.1 of Miller and Carter (2020) shows that this model is identifiable under the following constraints:

- $U^T U = V^T V = I_M$ (orthonormality),
- $\sigma_1 > \cdots > \sigma_M > 0$,
- the first non-zero entry in every column of U is positive,
- $\sum_{i=1}^I \alpha_i = 0$, $\sum_{i=1}^I u_{im} = 0$, and $\sum_{j=1}^J v_{jm} = 0$.

Many of the results presented in this section can easily be extended to cases with more complicated experimental designs (for example, adding row and column covariates), different link functions, and different outcome distributions. See Miller and Carter (2020) for more general GBM formulations.

S1.2 *Iteratively reweighted singular value decomposition (IRSVD)*

Weighted low-rank approximation. Given a matrix $Z \in \mathbb{R}^{I \times J}$, the weighted low-rank problem is to find a rank M matrix $X \in \mathbb{R}^{I \times J}$ that minimizes the weighted squared error between X and Z:

$$\sum_{i,j} W_{ij}(Z_{ij} - X_{ij})^2 \tag{S1.10}$$

where $W \in \mathbb{R}^{I \times J}$ is a matrix of known non-negative weights. When $W_{ij} = 1$ for all i and j, a solution can be found via the truncated singular value decomposition (SVD) of Z (Eckart and Young, 1936). Unfortunately, the general case cannot be reduced to an eigenvector problem unless $\text{rank}(W) = 1$. When the weights have been scaled to be in $[0, 1]$, Srebro and Jaakkola (2003) present the following iterative algorithm to find X:

$$X^{(t+1)} = \text{SVD}_M\big(W \circ Z + (1 - W) \circ X^{(t)}\big) \tag{S1.11}$$

where $\circ$ is the Hadamard product and $\text{SVD}_M(X)$ denotes the rank M truncated SVD of X. Tuzhilina and Hastie (2021) note that Equation S1.11 can be seen as a projected gradient descent step: since the gradient is

$$\nabla_X \frac{1}{2}\big\|\sqrt{W} \circ (Z - X)\big\|_F^2 = -W \circ (Z - X), \tag{S1.12}$$

a gradient step would be

$$X^{(t)} + \rho\left(W \circ (Z - X^{(t)})\right) \tag{S1.13}$$

which coincides with the argument of SVD_M in Equation S1.11 when the step size is $\rho = 1$. The projection back onto the set of rank M matrices is given by SVD_M. Further, Tuzhilina and Hastie (2021) show that the convergence rate can be improved by using the acceleration method of Nesterov (1983):

$$Q^{(t)} = X^{(t)} + \frac{t-1}{t+2}(X^{(t)} - X^{(t-1)}) \tag{S1.14}$$

$$X^{(t+1)} = \text{SVD}_M\big(Q^{(t)} + \rho(W \circ (Z - Q^{(t)}))\big). \tag{S1.15}$$

Approximating the Poisson bilinear model log-likelihood. We show that maximum likelihood estimation for X in the Poisson bilinear model in Equation 3.4 can be locally approximated by a weighted low-rank problem. Denoting $X_{ij} = (U\Sigma V^T)_{ij}$, the log-likelihood of X is

$$\ell(X) = \sum_{i=1}^{I}\sum_{j=1}^{J}\Big(Y_{ij}(\alpha_i + \beta_j + X_{ij}) - \exp(\alpha_i + \beta_j + X_{ij})\Big) + \text{const.} \tag{S1.16}$$

For now, we hold α_i and β_j at arbitrary fixed values. Differentiating with respect to X_{ij}, we have

$$\begin{aligned}\frac{\partial \ell}{\partial X_{ij}}(X) &= Y_{ij} - \exp(\alpha_i + \beta_j + X_{ij}),\\ \frac{\partial^2 \ell}{\partial X_{ij}^2}(X) &= -\exp(\alpha_i + \beta_j + X_{ij}).\end{aligned}$$

Suppose $\hat{X}$ is our current estimate of X, and denote $\hat{\mu}_{ij} = \exp(\alpha_i + \beta_j + \hat{X}_{ij})$. Then a second-order Taylor approximation at $\hat{X}$ yields

$$\begin{aligned}\ell(X) &\approx \ell(\hat{X}) + \sum_{i,j}\frac{\partial \ell}{\partial X_{ij}}(\hat{X})(X_{ij} - \hat{X}_{ij}) + \frac{1}{2}\sum_{i,j}\frac{\partial^2 \ell}{\partial X_{ij}^2}(\hat{X})(X_{ij} - \hat{X}_{ij})^2\\ &= \ell(\hat{X}) + \sum_{i,j}(Y_{ij} - \hat{\mu}_{ij})(X_{ij} - \hat{X}_{ij}) - \frac{1}{2}\sum_{i,j}\hat{\mu}_{ij}(X_{ij} - \hat{X}_{ij})^2\\ &= \text{const} - \frac{1}{2}\sum_{i,j}\hat{\mu}_{ij}\left(\frac{Y_{ij} - \hat{\mu}_{ij}}{\hat{\mu}_{ij}} + \hat{X}_{ij} - X_{ij}\right)^2\\ &= \text{const} - \frac{1}{2}\hat{\mu}_* \sum_{i,j} W_{ij}(Z_{ij} - X_{ij})^2\end{aligned}$$

where

$$\begin{aligned}\hat{\mu}_* &= \max_{ij}\hat{\mu}_{ij}\\ W_{ij} &= \hat{\mu}_{ij}/\hat{\mu}_*\\ Z_{ij} &= \hat{X}_{ij} + \frac{Y_{ij} - \hat{\mu}_{ij}}{\hat{\mu}_{ij}}.\end{aligned} \tag{S1.17}$$

Under this local approximation,

$$\operatorname*{arg\,max}_{X\,:\,\text{rank}(X)=M} \ell(X) \approx \operatorname*{arg\,min}_{X\,:\,\text{rank}(X)=M} \sum_{i,j} W_{ij}(Z_{ij} - X_{ij})^2. \tag{S1.18}$$

Thus, maximizing the likelihood of X in the Poisson bilinear model can be locally approximated by a weighted low-rank problem of the form in Equation S1.10, which can be solved using the Srebro and Jaakkola (2003) iteration in Equation S1.11.

Regularizing the singular values. It is known that GLMs with a log or logit link function can suffer from infinite MLEs when applied to sparse data (Correia *and others*, 2019). Similar issues can arise in the Poisson GBM; for example, consider a count matrix such that $Y_{11} = 0$ and $Y_{ij} = 1$ for all $i, j \neq 1$. An upper bound to the log-likelihood is $-IJ+1$ and when $M = 1$ this is achieved by taking $\alpha_i = 0$, $\beta_j = 0$, $u = (1, 0, 0, \ldots, 0) \in \mathbb{R}^I$, $v = (-1, 0, 0, \ldots, 0) \in \mathbb{R}^J$, and $\sigma \to \infty$. In other words, the leading singular value diverges to infinity. Although this choice of u and v do not satisfy the identifiability constraints, for any fixed σ constants can be rearranged to ensure that $\sum_{i=1}^I u_i = \sum_{j=1}^J v_j = 0$ (see Equation S1.32).

We have found it useful to place a prior on σ_m to ensure that this divergence does not occur. Specifically, we choose $\sigma_1, \ldots, \sigma_M \sim \text{Exponential}(\tau)$ independently, that is,

$$p(\sigma_m) = \tau \exp(-\tau \sigma_m). \tag{S1.19}$$

In this case, we aim to maximize the log posterior, or equivalently, the penalized log-likelihood:

$$\ell(X) - \tau \sum_{m=1}^{M} \sigma_m = \ell(X) - \tau \|X\|_* \tag{S1.20}$$

where $\|X\|_*$ denotes the nuclear norm, that is, the sum of the singular values of the matrix $X = U\Sigma V^\top$. In this setting, Tuzhilina and Hastie (2021) showed that a proximal gradient descent approach to Equation S1.20 yields an update

$$X^{(t+1)} = \text{SVD}_{M,\tau}(W \circ Z + (1 - W) \circ X^{(t)}) \tag{S1.21}$$

where $\text{SVD}_{M,\tau}$ denotes a rank M truncated SVD with the leading M singular values are soft-thresholded by τ. Specifically, if $A = U\text{diag}(\sigma_1, \ldots, \sigma_M)V^\top$ is the rank M SVD of a matrix A

then

$$\text{SVD}_{M,\tau}(A) := U\text{diag}((\sigma_1 - \tau)_+, \ldots, (\sigma_M - \tau)_+)V^\top \tag{S1.22}$$

where $(x)_+ = \max(x, 0)$.

Iteratively reweighting based on the local approximations. Unlike the standard weighted low-rank problem, however, the W and Z in Equation S1.18 depend on the current parameter estimates rather than being fixed. This suggests using an iterative algorithm in which the local approximation is sequentially updated based on the current estimates of $\hat{\alpha}$, $\hat{\beta}$, and $\hat{X} = \hat{U}\hat{\Sigma}\hat{V}^T$. Specifically, we propose alternating between the following two steps.

1. **Update intercepts.** Holding $\hat{\beta}$ and $\hat{X}$ fixed, for each i, the model reduces to a standard GLM with intercept α_i. In the Poisson case, there is a closed-form solution for maximizing with respect to α_i, which yields the update in Equation S1.29. Likewise, if one wishes to estimate β_j, this can be done in the same way by maximizing with respect to β_j, yielding Equation S1.30.

2. **Update latent factors.** Given estimates $\hat{\alpha}$ and $\hat{\beta}$, along with our current estimate $\hat{X}$, define W_{ij} and Z_{ij} by Equation S1.17. To obtain a new $\hat{X}$ that is closer to solving the local approximation in Equation S1.18, apply a generalized form of the Srebro and Jaakkola (2003) iteration by combining Equations S1.11 and S1.13, which yields the update in Equation S1.31. This can also be modified to use Nesterov acceleration as in Equation S1.14.

Initialization. Using a good choice of initialization is important for fast and reliable convergence. If we were to initialize $\hat{X} = 0$, then after the first update to the intercepts, the weights would be

$$W_{ij} = \exp(\hat{\alpha}_i + \hat{\beta}_j), \tag{S1.23}$$

which makes W a rank 1 matrix. When $\text{rank}(W) = 1$, it turns out that the weighted low-rank problem can be solved in one step by reducing to a standard SVD. Thus, it is preferable to solve this initial weighted low-rank problem exactly rather than just performing one iteration of Equation S1.31. The exact solution is derived as follows.

Proposition 1 If $W = d_1 d_2^T$ where $d_1 \in \mathbb{R}^I$, $d_2 \in \mathbb{R}^J$, and $W_{ij} > 0$ for all i, j, then

$$X = \sqrt{D_1^{-1}}\,\text{SVD}_M\big(\sqrt{D_1} Z \sqrt{D_2}\big)\sqrt{D_2^{-1}} \tag{S1.24}$$

minimizes Equation S1.10 subject to $\text{rank}(X) \leqslant M$, where $D_1 = \text{diag}(d_1)$ and $D_2 = \text{diag}(d_2)$.

Proof. The proof is due to Razenshteyn *and others* (2016). Since $W_{ij} = d_{1i} d_{2j}$, we have

$$\sum_{i,j} W_{ij}(Z_{ij} - X_{ij})^2 = \big\|\sqrt{D_1}(Z - X)\sqrt{D_2}\big\|_F^2 = \big\|\sqrt{D_1} Z \sqrt{D_2} - A\big\|_F^2. \tag{S1.25}$$

where $A = \sqrt{D_1} X \sqrt{D_2}$. Let $\mathcal{R}_M = \{A \in \mathbb{R}^{I \times J} : \text{rank}(A) \leqslant M\}$. By the Eckart–Young theorem, $A^* = \text{SVD}_M(\sqrt{D_1} Z \sqrt{D_2})$ minimizes Equation S1.25 over $A \in \mathcal{R}_M$. Since $W_{ij} > 0$ implies $d_{1i} > 0$ and $d_{2j} > 0$, it follows that $X \mapsto \sqrt{D_1} X \sqrt{D_2}$ is a bijection from $\mathcal{R}_M$ to itself. Thus, $X^* = \sqrt{D_1^{-1}} A^* \sqrt{D_2^{-1}}$ minimizes Equation S1.25 over $X \in \mathcal{R}_M$. □

Applying Proposition 1 suggests using an initial estimate of

$$\hat{X} = \sqrt{D_1^{-1}}\text{SVD}_M(\sqrt{D_1} Z \sqrt{D_2})\sqrt{D_2^{-1}} = \text{SVD}_M\big((Y - W)/\text{sqrt}(W)\big)/\text{sqrt}(W) \tag{S1.26}$$

where sqrt and / denote entry-wise square root and entry-wise division, respectively. In practice, it is necessary to ensure that $\hat{X}$ does not contain extreme values. For this reason, we replace the singular values in (S1.26) with singular values drawn from an exponential distribution.

Interestingly, note that since $W = \hat{\mu}$, the entries of $(Y - W)/\text{sqrt}(W)$ coincide with the Pearson residuals under a Poisson model. Thus, the derivation of Equation S1.26 provides some theoretical justification for using PCA on the Pearson residuals, as done by scTransform (Hafemeister and Satija, 2019), by thinking of it as an approximation to GBM parameter estimation. However, the

assumption that W is rank 1 (or close to it) is unlikely in cases where there is a large amount of latent structure. Thus, in many cases of interest, this will only be a very rough approximation.

S1.3 *Step-by-step estimation algorithm.*

Based on the above derivations, in this section we provide a step-by-step description of the estimation algorithm.

- **Initialize.** Set $\hat{\beta}_j \leftarrow \log(\sum_i Y_{ij})$, $\hat{\alpha}_i \leftarrow \log(\sum_j Y_{ij}) - \log(\sum_j \exp(\hat{\beta}_j))$, $W_{ij} \leftarrow \exp(\hat{\alpha}_i + \hat{\beta}_j)$. Draw $\sigma_1, \ldots, \sigma_M \sim \text{Exponential}(1/10)$, and define $\hat{U}$ and $\hat{V}$ to be the left and right singular vectors of

$$\text{SVD}_M\big((Y - W)/\text{sqrt}(W)\big)/\text{sqrt}(W) \tag{S1.27}$$

  Then set

$$\hat{X}^{(0)} = \hat{U}\text{diag}(\sigma_{(1)}, \ldots, \sigma_{(M)})\hat{V}^\top \tag{S1.28}$$

  where $\sigma_{(i)}$ denotes the i-th order statistic.

- Iterate the following steps until convergence. On iteration $t \geqslant 1$,

  1. **Update intercepts.**

$$\hat{\alpha}_i \leftarrow \log\Big(\sum_{j=1}^{J} Y_{ij}\Big) - \log\Big(\sum_{j=1}^{J} \exp(\hat{\beta}_j + \hat{X}_{ij}^{(t)})\Big), \tag{S1.29}$$

$$\hat{\beta}_j \leftarrow \log\Big(\sum_{i=1}^{I} Y_{ij}\Big) - \log\Big(\sum_{i=1}^{I} \exp(\hat{\alpha}_i + \hat{X}_{ij}^{(t)})\Big). \tag{S1.30}$$

  2. **Update latent factors.** Compute $\hat{\mu}_{ij} \leftarrow \exp(\hat{\alpha}_i + \hat{\beta}_j + \hat{X}_{ij})$, $\hat{\mu}_* \leftarrow \max_{ij} \hat{\mu}_{ij}$, $W_{ij} \leftarrow \hat{\mu}_{ij}/\hat{\mu}_*$, $Z_{ij} \leftarrow \hat{X}_{ij} + (Y_{ij} - \hat{\mu}_{ij})/\hat{\mu}_{ij}$, and

$$\begin{aligned} Q^{(t)} &\leftarrow \hat{X}^{(t)} + \frac{t-1}{t+2}(X^{(t)} - X^{(t-1)}) \\ \hat{X}^{(t+1)} &\leftarrow \text{SVD}_{M,\tau}\big(Q^{(t)}\big). \end{aligned} \tag{S1.31}$$

- **Output.** Return the estimates $\hat{\alpha}_i$, $\hat{\beta}_j$, $\hat{U}$, $\hat{\Sigma}$, and $\hat{V}$ such that $\hat{X} = \hat{U}\hat{\Sigma}\hat{V}^T$.

For the convergence criterion, we terminate the algorithm when the relative change in log-likelihood is below a specified tolerance (default 10^{-4}) or after a specified maximum number of iterations (default 100). As a default, we set $M = 20$. We find that a step size of $\rho = 1$ works well, but that the rate of convergence can be improved by using an adaptive scheme where ρ is increased by a multiplicative factor of 1.05 when the log-likelihood increases and is decreased by a multiplicative factor of 1/2 when the log-likelihood decreases.

Identifiability. Note that the constraints $U^\top U = I_M$, $V^\top V = I_M$, and $\sigma_1 > \ldots > \sigma_M > 0$ are enforced in each step of the algorithm by definition of the singular value decomposition; we have found that this is important for the performance of the algorithm. The remaining constraints account for invariance under certain multiplicative or additive transformations, and we have found that it is sufficient to enforce these in a post-hoc step. If the first non-zero entry in a column of U is negative, then we can multiply the entire column by -1 and the corresponding column of V by -1. For ensuring that columns of U and V sum to 0, the following is applied:

$$\begin{aligned}
\overline{u}_m &\leftarrow \frac{1}{I}\sum_{i=1}^{I} u_{im} \\
\beta_j &\leftarrow \beta_j + \sum_{m=1}^{M} \sigma_m \overline{u}_m v_j \\
U_{\cdot m} &\leftarrow U_{\cdot m} - \overline{u}_m \\
\overline{v}_m &\leftarrow \frac{1}{J}\sum_{j=1}^{J} v_{jm} \\
\alpha_i &\leftarrow \alpha_i + \sum_{m=1}^{M} \sigma_m \overline{v}_m u_i \\
V_{\cdot m} &\leftarrow V_{\cdot m} - \overline{v}_m \\
\beta &\leftarrow \beta + \frac{1}{I}\sum_{i=1}^{I} \alpha_i \\
\alpha &\leftarrow \alpha - \frac{1}{I}\sum_{i=1}^{I} \alpha_i
\end{aligned} \tag{S1.32}$$

Post-hoc ordering of latent factors. Unlike standard PCA, the estimated singular value $\hat{\sigma}_1$ do not necessarily correspond to the factor with the largest deviance explained (Van Eeuwijk, 1995). For example, in the previous section we demonstrated an example Y where a singular value was driven by a single entry in the count matrix. Thus, instead of ranking the factors in order of $\hat{\sigma}_m$, our final step is to rearrange factors in order of decreasing deviance (with respect to the full model). Specifically, let

$$\tilde{\mu}^{(m')} = \exp\left(\hat{\alpha}_i + \hat{\beta}_j + \sum_{m \neq m'} \hat{\sigma}_m \hat{u}_{im} \hat{v}_{jm}\right) \tag{S1.33}$$

be the fitted mean with factor m' removed. Then define

$$D_{m'} = \ell(\hat{\mu}) - \ell(\tilde{\mu}^{(m')}) \tag{S1.34}$$

to be the difference between the full model log-likelihood and the log-likelihood with factor m' removed. Finally, permute the columns of U and V to ensure that the factors appear in decreasing order of D_m.

S1.4 *Computational complexity.*

0. **Initialization.** The rank M truncated SVD takes $O(IJM)$ time (Halko *and others*, 2011). The other operations only take $O(IJ)$ time, so initialization is $O(IJM)$ altogether.

1. **Update intercepts.** Each $\hat{\alpha}_i$ update takes $O(J)$ time, and each $\hat{\beta}_j$ update takes $O(I)$ time. Thus, the entire step requires $O(IJ)$ time.

2. **Update latent factors.** The entry-wise operations are $O(IJ)$ and the projection (rank M truncated SVD) takes $O(IJM)$ time (Halko *and others*, 2011).

Thus, the overall runtime for one iteration of IRSVD is $O(IJM)$. This is faster than the Fisher scoring algorithm of Miller and Carter (2020), which is $O(IJM^2)$ per iteration. The quadratic complexity in M can be practically significant since M is often chosen to be around 20-50. Further,

IRSVD is simpler than the Miller and Carter (2020) algorithm, making it easier to implement and yielding a smaller constant.

S1.5 *Cluster cohesion index*

Given estimates $\hat{v}_{jm}$ and corresponding standard errors $\mathrm{se}(\hat{v}_{jm})$, we aim to quantitatively analyze the stability of a given clustering of cells. Let $c_1, \ldots, c_J \in \{1, \ldots, K\}$ be assignments of the J cells to K clusters. To compute the *cluster cohesion indices* (CCIs), we repeat the following steps n times (by default, $n = 100$):

1. Draw $\tilde{v}_{jm} \sim \mathcal{N}(\hat{v}_{jm}, \mathrm{se}(\hat{v}_{jm})^2)$ for $j = 1, \ldots, J$, $m = 1, \ldots, M$.
2. Apply a clustering algorithm to the rows of $\tilde{V} = [\tilde{v}_{jm}]$ to obtain new cluster assignments $\tilde{c}_1, \ldots, \tilde{c}_J$.
3. For each pair of clusters $k, k' \in \{1, \ldots, K\}$, compute the fraction

$$f_{k,k'} = \frac{1}{|S_{k,k'}|} \sum_{(j,j') \in S_{k,k'}} \mathrm{I}(\tilde{c}_j = \tilde{c}_{j'}) \tag{S1.35}$$

where $S_{k,k'}$ is the set of pairs $j, j' \in \{1, \ldots, J\}$ such that $j \neq j'$, $c_j = k$, and $c_{j'} = k'$.

In Equation S1.35, $\mathrm{I}(\cdot)$ denotes the indicator function. Then, for each cluster k, the CCI is defined as the mean (across the n repetitions) of the fraction $f_{k,k}$. Likewise, the inter-cluster cohesion index (inter-CCI) for clusters k and k' is defined as the mean of $f_{k,k'}$. That is, the inter-CCI is the mean of the fraction of all pairs of points j and j' that are in the same cluster after resampling, out of all pairs j and j' that were originally in clusters k and k', respectively.

The interpretation is that a low CCI indicates that this cluster could be an artifact of sampling variability. Likewise, a high inter-CCI for clusters k and k' indicates that the separation of these two clusters may be an artifact of sampling variability. Whenever possible, the clustering algorithm in step 2 should be the same as the one used to obtain the original assignments $c_1, \ldots, c_J$.

To characterize the CCI under the null of no latent variability we replace $\tilde{v}_{jm}$ in step 1 with $v_{jm,\text{null}} \sim \mathcal{N}(0, \text{se}(\hat{v}_{jm})^2)$ for $j = 1, \ldots, J$, $m = 1, \ldots, M$. Then we perform steps 1-3 above for $n = 100$ repetitions, and compute the 95th percentile of the resulting $f_{k,k}$ values to get the dashed blue lines in Figure 4b.

S1.6 *Semi-simulated hybrid T/B cells*

We used real data to construct a semi-simulated dataset with cells that lie on a "gradient" between two different cell types. Beginning with a dataset of B cells and naive T cells from the *DuoClustering2018* package (Duò *and others*, 2018), we simulated hybrid cells by combining a fraction of the genes from each cell type. Specifically, we used the following procedure to generate a dataset with $I = 6168$ genes and $J = 5000$ hybrid cells, where each cell is generated as follows:

1. Randomly select one B cell and one naive T cell.
2. Sample $I' \sim \text{Unif}\{1, \ldots, I\}$ and randomly choose a subset of I' genes.
3. Construct a simulated cell such that the counts for the I' randomly chosen genes are equal to those of the memory T cell, and the remaining $I - I'$ genes counts are equal to those of the naive T cell.

We then tested the ability of each method to capture the biological gradient, which is mathematically represented by the mixture proportion I'/I (Figure S16). We quantified performance by computing the squared correlation r^2 between the mixture proportion and each embedding dimension. We found that the first component of scGBM outperformed all competing methods except for Log+PCA ($r^2 = 0.81$ vs $r^2 = 0.755$). However, after applying UMAP to Log+PCA (the standard approach for visualization), two spurious clusters are created that would suggest two discrete cell types as opposed to a continuum.

An additional strength of scGBM is that the low-dimensional embeddings are directly inter-

pretable. Specifically, a unit change along the x-axis of the scGBM plot in Figure S16 corresponds to a log fold-change of 1 with respect to the linear combination of genes defined by the first factor loadings. Because scGBM provides a p-value for each element u_{i1}, one can visualize the important genes in each factor using a volcano plot (Figure S17). Theoretical calculations (in the following paragraph) suggest that genes with absolute weight $|u_{i1}|$ greater than $2/\sqrt{I} \approx 0.025$ should be considered as relevant to the factor. For example, *FCRL2* ($u = -0.035$, $p = 1.28 \times 10^{-14}$) is known to be preferentially expressed by B cells (Li *and others*, 2014), whereas *TCEA3* ($u = 0.028$, $p = 1.53 \times 10^{-15}$) is reported to be enhanced in naive and memory T cells (Uhlén *and others*, 2015, Human Protein Atlas, www.proteinatlas.org).

We now describe our approach to obtain the $2\sqrt{I}$ bound. Under the null model, the first estimated factor $\hat{U}_1 \in \mathbb{R}^I$ is uniformly distributed on the surface of the ball $S^{I-1} := \{x \in \mathbb{R}^I : \|x\|_2 = 1\}$. A heuristic cutoff is to choose genes such that their weight is greater than what would be expected under this null model. In this case $\hat{U}_1$ has same distribution as a multivariate standard normal random variable $Z \sim \mathcal{N}(0, \mathrm{I})$ that has been projected onto S^{I-1}:

$$\hat{U}_1 = \frac{Z}{\|Z\|_2}. \tag{S1.36}$$

By the law of large numbers and the continuous mapping theorem,

$$\frac{\sqrt{I}}{\|Z\|_2} \xrightarrow{a.s.} 1 \tag{S1.37}$$

so the distribution of $\hat{U}_1$ can be approximated by the distribution of (suitably scaled) folded normal distributions

$$\frac{1}{\sqrt{I}} \left(|Z_1|, \ldots, |Z_p|\right) \tag{S1.38}$$

Applying standard results shows that

$$\mathbb{P}(\hat{U}_{11} > 2/\sqrt{I}) \leqslant 0.05 \tag{S1.39}$$

and

$$\mathbb{E}\|\hat{U}_1\|_\infty \leqslant \sqrt{\frac{2\log(2I)}{I}}. \tag{S1.40}$$

This justifies the use of $2/\sqrt{I}$ or the more stringent $\sqrt{2\log(2I)/I}$ cutoff to consider a gene as relevant to a factor.

S1.7 *Controlling for known batches*

When there are known batches, the model can be adjusted to have a gene-level intercept for each batch:

$$\log(\mu_{ij}) = \alpha_{ib_j} + \beta_j + \sum_{m=1}^{M} \sigma_m u_{im} v_{jm} \tag{S1.41}$$

where $b_j \in \{1, \dots, B\}$ is the index of the batch that cell j belongs to, and α_{ib} is the intercept for gene i and batch b. Estimation in this model is the same as before, except Equation S1.29 is replaced with

$$\hat{\alpha}_{ib} = \log\Big(\sum_{j\,:\,b_j=b} Y_{ij}\Big) - \log\Big(\sum_{j\,:\,b_j=b} \exp(\hat{\beta}_j + \hat{X}_{ij})\Big). \tag{S1.42}$$

S2. Data and software availability

We briefly describe the single-cell datasets used in the paper. Unless otherwise specified, all single-cell data was processed using Seurat (Satija *and others*, 2015). When it is stated that variable genes were selected, this means that the function `FindVariableFeatures()` was used to select the genes as input to scGBM.

- The ERCC data was downloaded from the 10X genomics website. The count matrix is available in the folder "Gene / cell matrix (filtered)."

- The 10X immune cell data (Zheng *and others*, 2017), including the Naive T cells and memory T cells used in Figure S16, were downloaded from the *DuoClustering2018* R package (Duò *and others*, 2018).

- The COVID-19 Atlas (Wilk *and others*, 2020) was downloaded as a Seurat object from www.covid19cellatlas.org.

- The 10X mouse brain data was downloaded from the Bioconductor package *TENxBrain* (Lun and Morgan, 2020).
- The Wu *and others* (2021) data was downloaded from the Gene Expression Omnibus (GEO) database (GSE176078).

scGBM is available for download as an R package at https://github.com/phillipnicol/scGBM. The repository also includes scripts for replicating the figures.

S3. Technical details of simulations

S3.1 *Single marker genes simulation*

The single marker genes simulation used $I = 1000$ genes, $J = 1000$ cells, and four cell types A, B, C, D. If cell j is of type t, then the observed count is generated as $Y_{ij} \sim \text{Poisson}(\mu_{i,t})$ where $\mu_{i,t}$ is defined as follows:

1. $\mu_{1,A} = 10$, $\mu_{1,B} = 1$, $\mu_{1,C} = \mu_{1,D} = 100$.
2. $\mu_{2,A} = \mu_{2,B} = \mu_{2,C} = 1$, $\mu_{2,D} = 50$.
3. $\mu_{i,t} = 1$ for all $3 \leqslant i \leqslant 1000$ and all $t \in \{A, B, C, D\}$.

For the sensitivity analysis (Figure S3), we excluded cell type B and focused on changing the number of A type cells, the value of $\mu_{1,A}$ and the value of $\mu_{2,D}$. For each method, we compared the estimated gene loading vectors to $e_1 \in \mathbb{R}^I$ (the first standard basis vector). The mean of cell type A and the remaining cell types are completely separated in the direction of e_1. Specifically, we define *separation* as

$$\max_{1 \leqslant m \leqslant M} \left| \hat{U}_{m,1} / (\max_{j \neq 1} \hat{U}_{m,j}) \right| \tag{S3.43}$$

where $\hat{U} \in \mathbb{R}^{I \times M}$ is the gene loading matrix for PCA/scGBM. Values greater than 1 indicate that some signal was identified.

S3.2 *Subsampling 10X immune cells*

To create heterogeneous cell type size distribution within the 10X immune cells (Zheng *and others*, 2017), we randomly sub-sampled all cell types other than CD14 monocytes to have at most 100 cells. We kept all of the 600 original CD14 monocytes.

S4. Supplementary Figures and Tables

Dataset	# of cells	scGBM-proj	scGBM-full	GLM-PCA (Fisher)	GLM-PCA (AvaGrad)	GLM-PCA (SGD)	NewWave
10X immune cells	3,994	0.14 min	1.68 min	55.72 min	1.58 min	1.72 min	2.95 min
COVID-19 Atlas	44,721	1.89 min	22.77 min	468.24 min	19.52 min	20.66 min	35.31 min
10X mouse brain	1,308,421	57.67 min	782.40 min	9878.20 min*	811.09 min	505.82 min	>2907 min

Table S1. Runtime comparison of scGBM, GLM-PCA, and NewWave on three datasets: 10X immune cells, COVID-19 Atlas, and 10X mouse brain; see Section S2 for dataset references. For each dataset, $I = 1000$ variable genes were selected using the Seurat package (Satija *and others*, 2015). Each method was forced to run for 100 iterations. For scGBM-proj, we used subsamples of sizes 400, 4,000, and 100,000 on the three datasets, respectively, and 64 cores were allocated for parallel processing. For GLM-PCA (SGD), the minibatch size was set equal to the subsample size used by scGBM-proj. For GLM-PCA, the learning rate (for Avagrad and SGD) and the penalty (for Fisher scoring) were set sufficiently small (or large) to prevent divergence. Latent factor dimensionality of $M = 20$ was used for all algorithms. When applying NewWave to the 10X brain data, the job timed out after 2907 minutes. *The runtime for GLM-PCA (Fisher) on the 10X mouse brain data was estimated by extrapolating the time taken for 10 iterations.

**Method**	**Cluster consistency**
scGBM	0.988
APR+PCA	0.639
Log+Scale+PCA	0.683
SCT+PCA	0.571
Log+PCA	0.767
GLM-PCA (SGD)	0.542

Table S2. The median cluster consistency of monocytes across five count splitting iterations. For a clustering c of monocytes from Y_1, and another clustering $\tilde{c}$ of monocytes from Y_2, the cluster consistency is defined as $\left(\sum_{ij} I(c_i = c_j)I(\tilde{c}_i = \tilde{c}_j)\right)/\left(\sum_{ij} I(c_i = c_j)\right)$. Here, the sums are over cells i and j that were labeled as monocytes. Because Y_1 and Y_2 are independent and identically distributed, it is symmetric to choose either c or $\tilde{c}$ in the denominator.

**Method**	**Cluster consistency**	**# clusters (full data)**	**# clusters (subsampled)**
scGBM	0.739	21	16
APR+PCA	0.526	23	18
Log+Scale+PCA	0.624	25	19
SCT+PCA	0.407	27	21
Log+PCA	0.546	24	14
GLM-PCA (SGD)	0.542	23	28

Table S3. Using the cell type labels provided by the authors of the COVID-19 data (Wilk *and others*, 2020), we subsample such that there are 10,339 CD14 monocytes and at most 100 cells of each other type. We then compute the cluster consistency (using the same formula as in Table S2) between the clusterings based on the full data and the subsampled data.

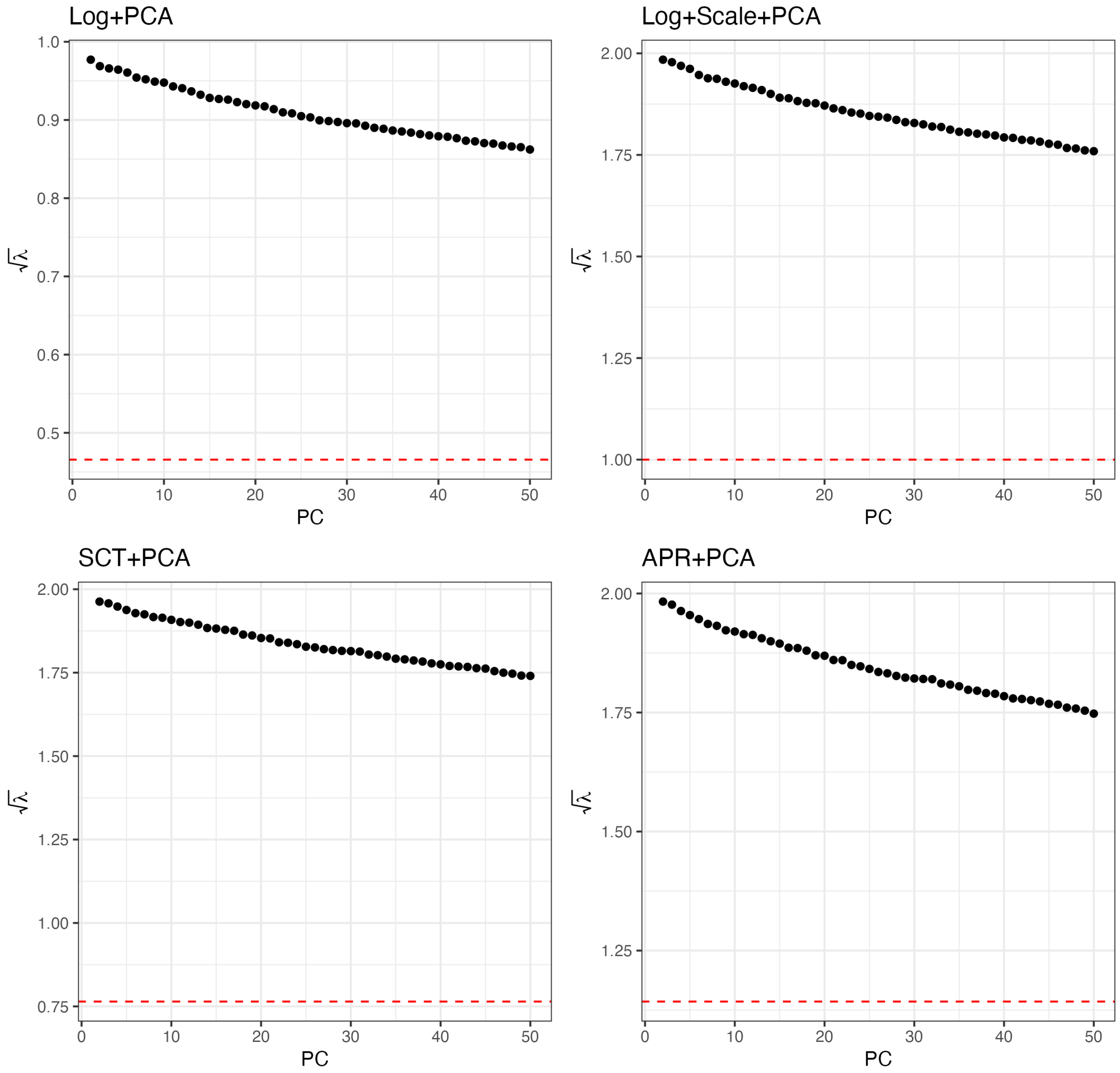

Fig. S1. For each method, we plot the standard deviation explained by each PC (black points), computed as $\sqrt{\lambda}$ where λ is the corresponding eigenvalue. The red dashed line is the standard deviation of gene 1 (the signal gene) after normalization. The fact that the red dashed line is below the leading $\sqrt{\lambda}$ values shows that the signal in gene 1 is undetectable by PCA. Because Log+PCA+Scale scales each gene to have unit variance, the dashed red line would lie below the leading eigenvalues irrespective of the strength of the marker gene.

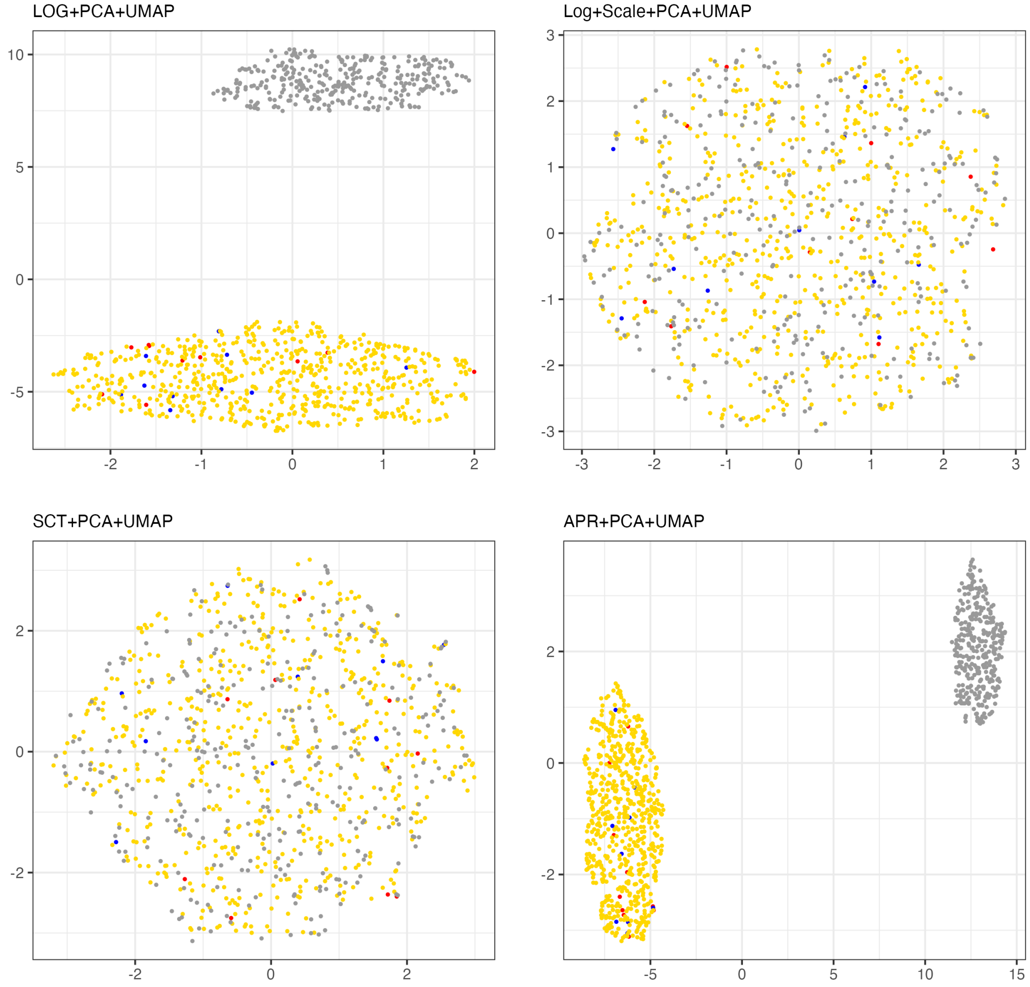

Fig. S2. Results of applying UMAP to the top 10 principal components from the PCA-based methods, for the single marker genes simulation.

[*Received August 1, 2010; revised October 1, 2010; accepted for publication November 1, 2010*]

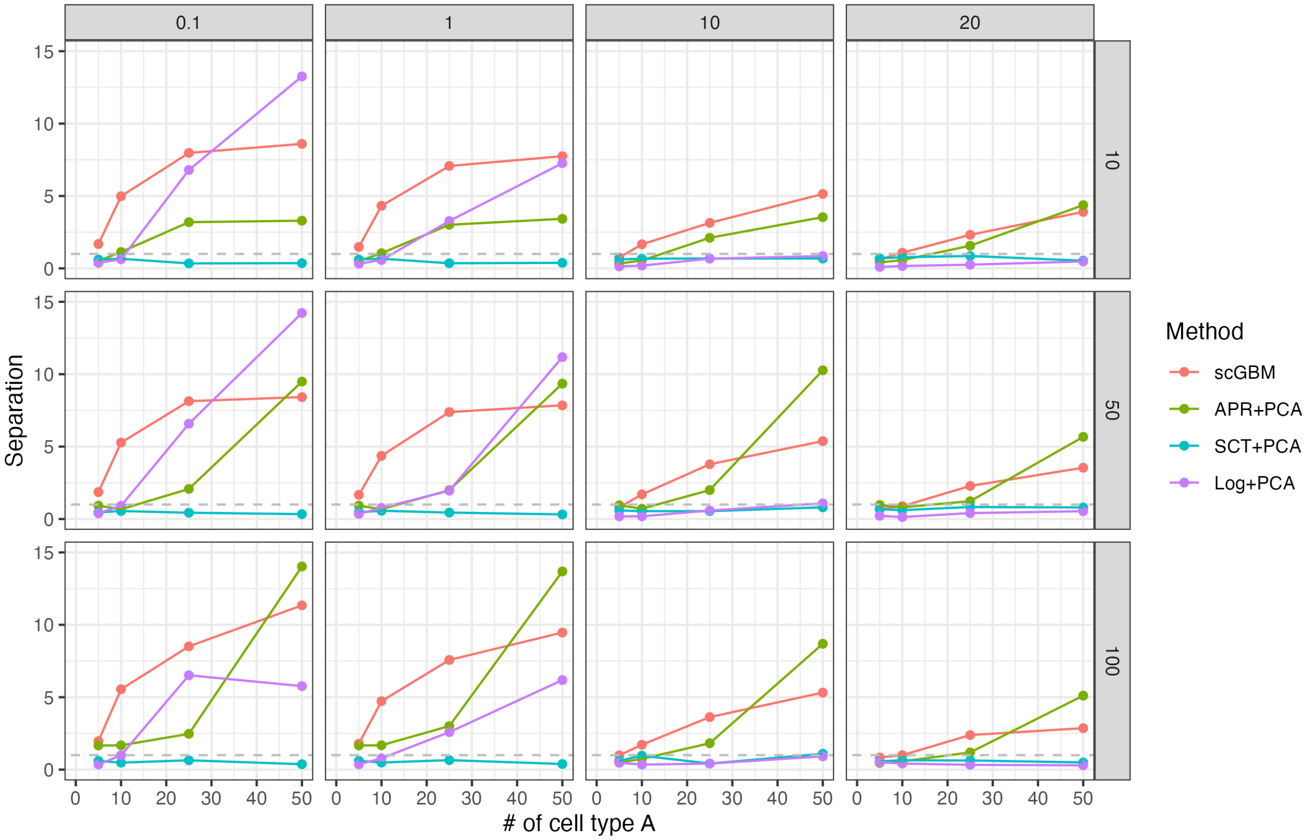

Fig. S3. Sensitivity analysis of single marker genes simulation as described in Section S3. Higher separation (defined in Section S3) indicates that the method is better able to distinguish the cell types. The column values indicate the mean expression of the marker gene for the rare cell type A and the row values indicate the mean expression of the marker gene for the common cell type.

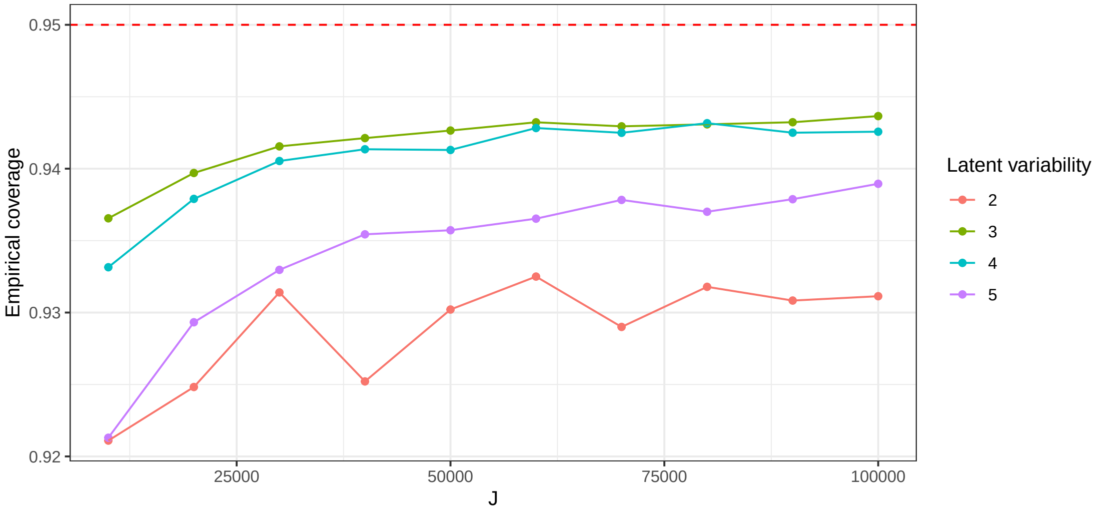

Fig. S4. Empirical coverage of 95% confidence intervals across different simulation settings for J and latent variability $\kappa = \sigma_1/\sigma_M$. Confidence intervals were formed using a normal approximation: $\hat{v}_{jm} \pm 1.96\,\mathrm{se}(\hat{v}_{jm})$. The empirical coverage is the fraction of times the confidence interval contained the true v_{jm}. Points shown are the median across 20 replicates.

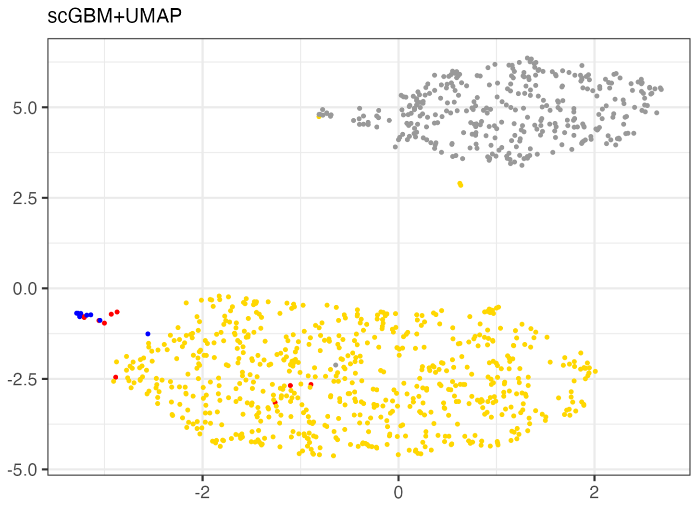

Fig. S5. Result of applying UMAP to the top 10 scGBM scores for the single marker genes simulation.

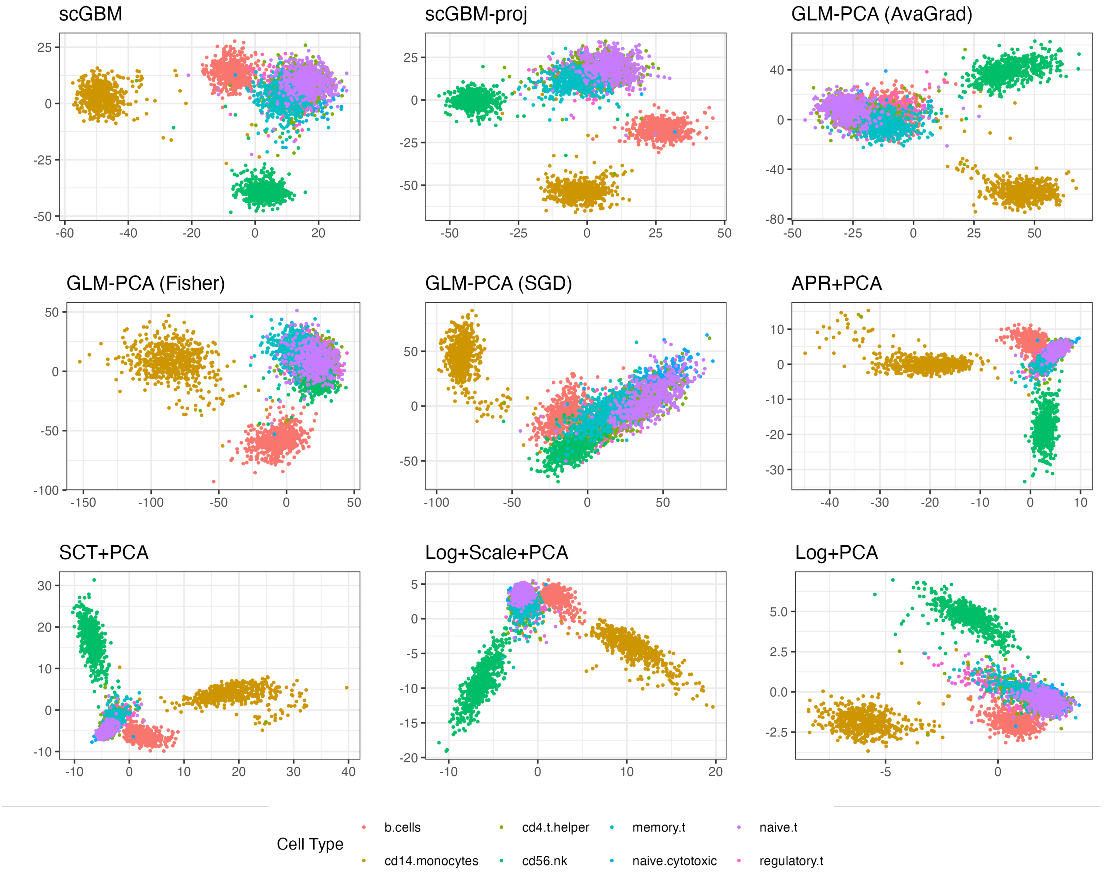

Fig. S6. The final embeddings estimated by each of the methods on the 10X immune cell data. All methods show strong separation in the CD14 monocytes and CD56 NK. However, for the PCA-based methods, these two populations appear "stretched out" compared to scGBM and GLM-PCA.

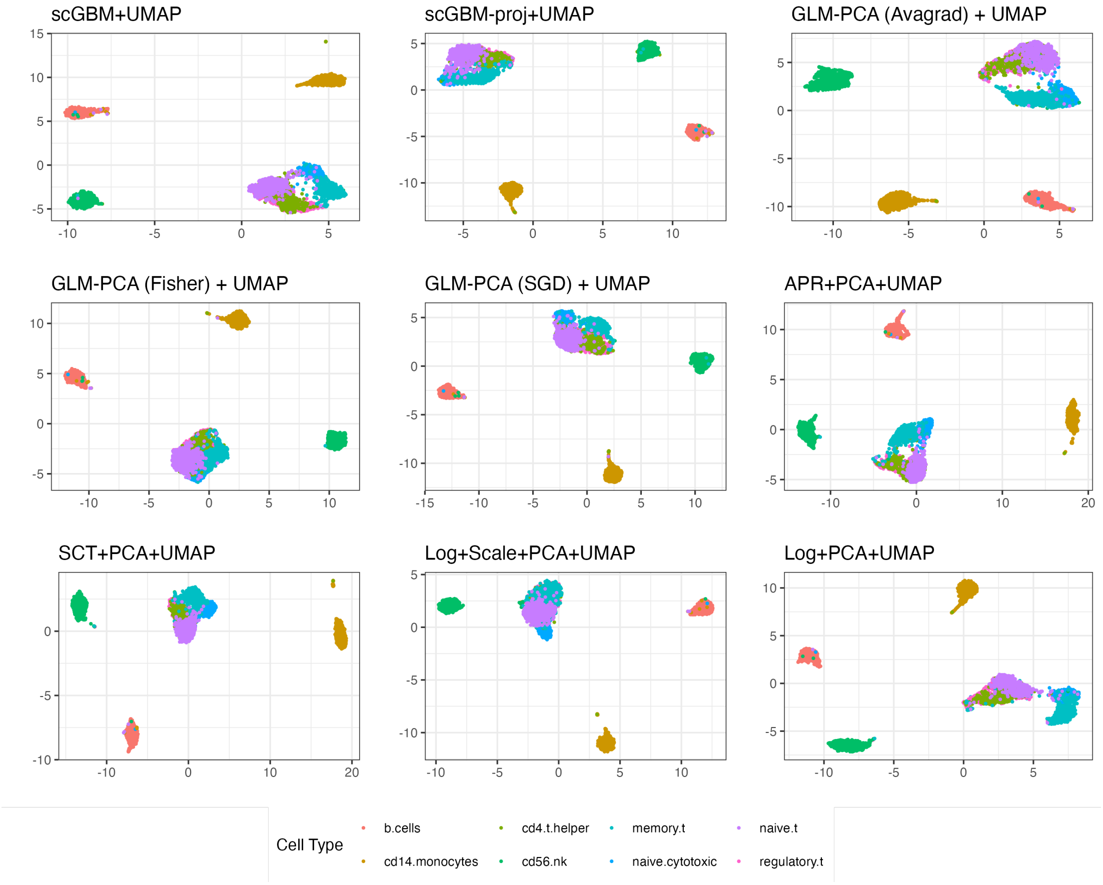

Fig. S7. The UMAP visualization for each of the methods on the 10X immune cell data. All of the embeddings appear qualitatively similar, with minor differences in the separation of the T cell subtypes.

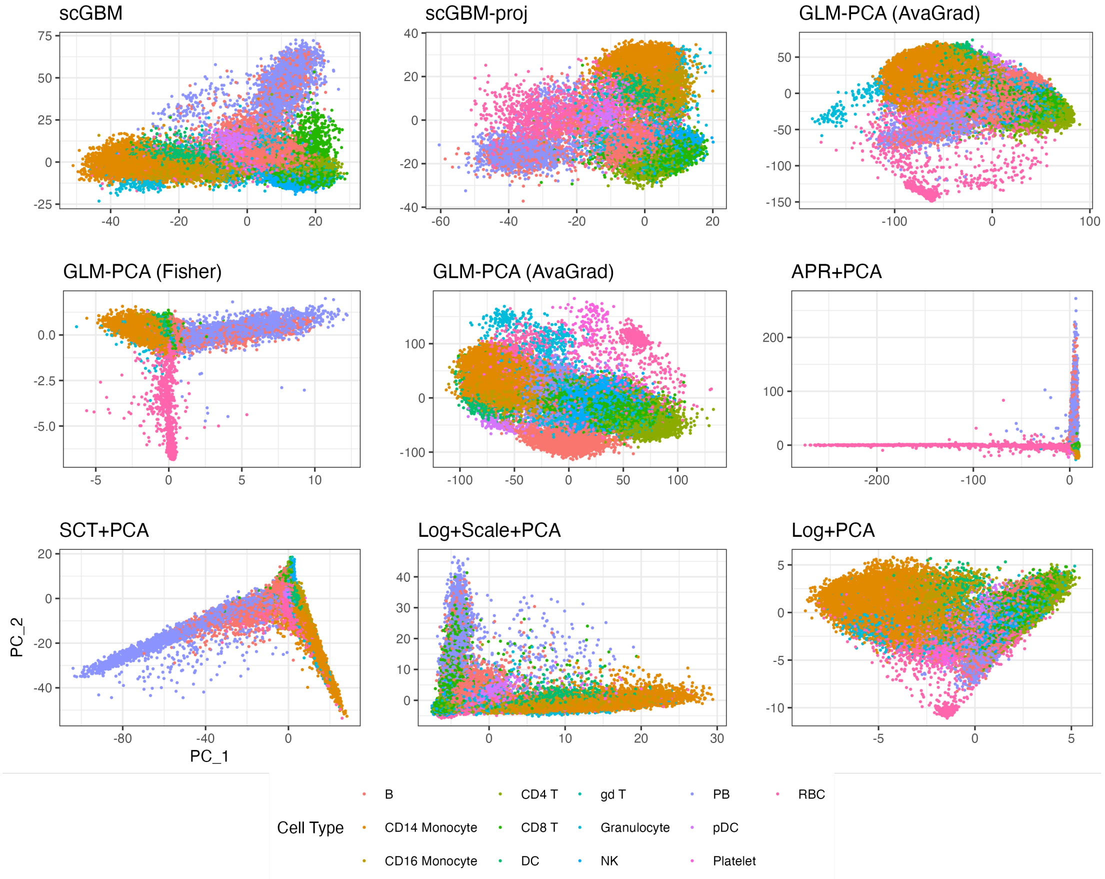

Fig. S8. The final embeddings estimated by each of the methods on the COVID-19 atlas data. As in the 10X immune cell data, some cell subpopulations appear stretched out by PCA. The most extreme example is APR+PCA where the red blood cells (RBC) dominate the entire first PC and plasmacytoid dendritic cells (pDC) dominate the second PC.

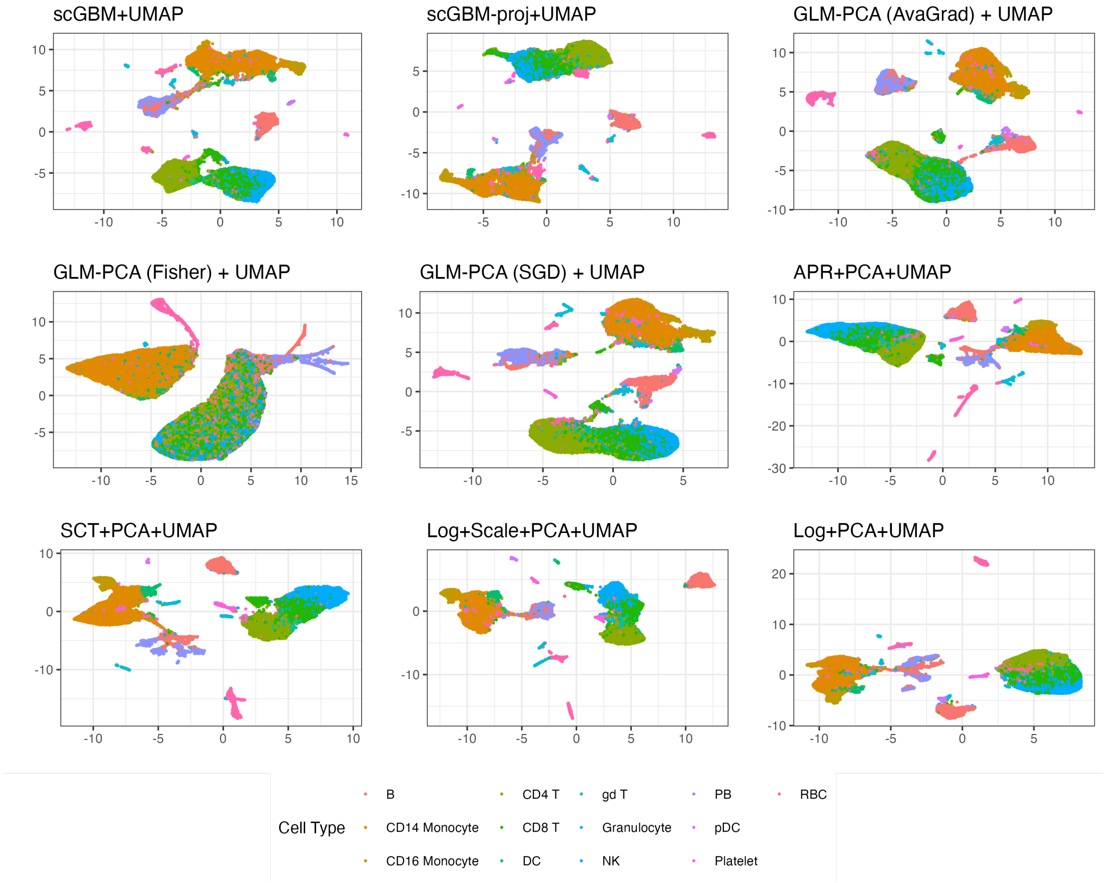

Fig. S9. The UMAP visualization for each of the methods on the COVID-19 atlas data. The embeddings appear qualitatively similar, with the exception of GLM-PCA (Fisher), which shows noticably less separation among the different cell types.

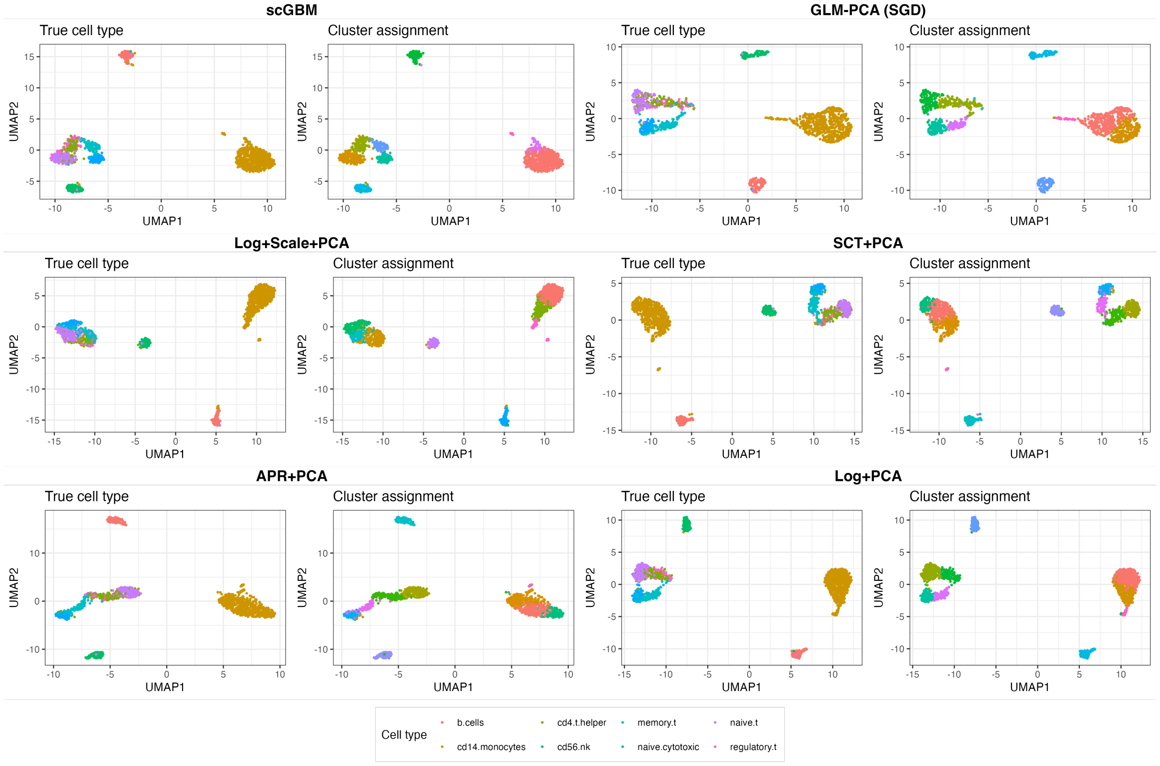

Fig. S10. UMAP embeddings for the imbalanced 10X immune cell data, using each dimension reduction method. For each method, the left plot colors the cells by true cell type whereas the right plot indicates cluster assignment obtained by running the Louvain algorithm with resolution 0.8, as in Figure 3b. The total number of clusters estimated was 8 for scGBM and GLM-PCA; 9 for Log+PCA and Log+Scale+PCA; and 10 for SCT+PCA and APR+PCA.

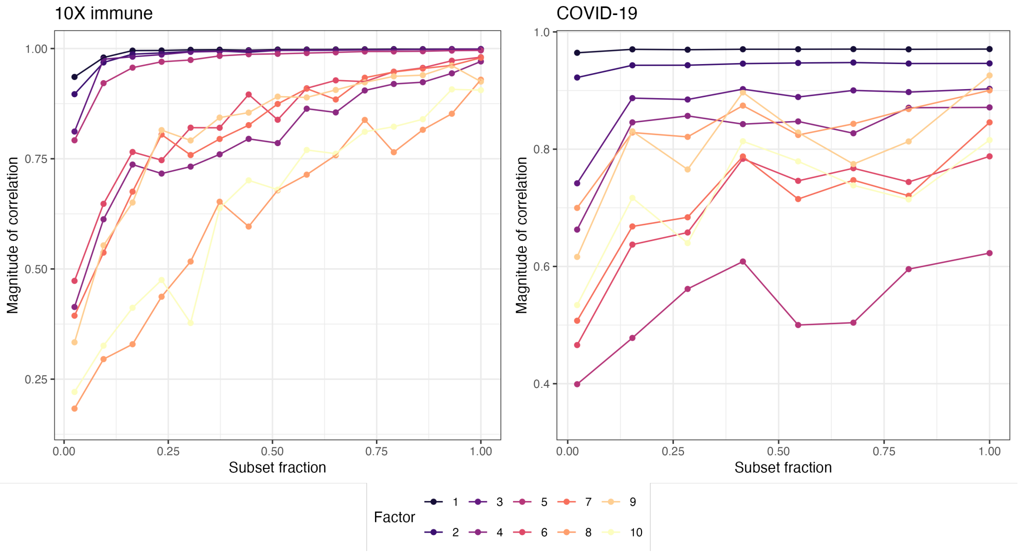

Fig. S11. Testing the accuracy of the projection method. We first applied scGBM-full to the 10X immune cell dataset ($J = 3{,}994$) to estimate V. Then for subsets of various sizes, we used scGBM-proj to estimate $\hat{V}$, and for each $m = 1, \ldots, 10$, computed the maximum absolute correlation between the m-th column of $\hat{V}$ and any column of V. The points shown represent the mean of this correlation over 10 runs of scGBM-proj. As the subset fraction increases, scGBM-proj agrees more closely with scGBM-full, with 10-15% being sufficient to accurately capture the largest factors. Running the same analysis on the COVID-19 Atlas dataset ($J = 44{,}721$), we find that scGBM-proj provides similar results for subsample sizes from around 25% to 100%, but that the correlation with scGBM-full remains somewhat lower for the smaller factors. The 10X mouse brain dataset was omitted due to the computational burden of numerous runs required.

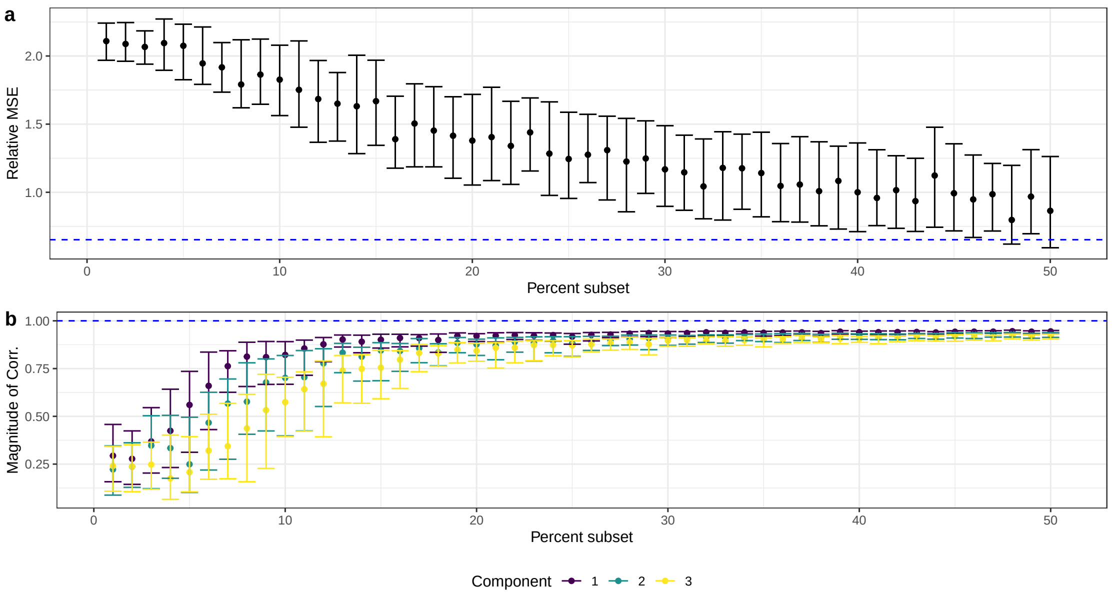

Fig. S12. Testing the accuracy of the projection method using simulated data with $I = 1000$, $J = 10000$, and $M = 10$. **a.** Relative MSE between ground truth and scGBM-proj estimate of V as a function of subsample size used. The points are the median across 100 trials and the error bars represent the interquartile range. The dashed blue line is the median of the relative MSE for scGBM-full. **b.** Absolute value of the correlation between ground truth and scGBM-proj estimate of V as a function of subsample size. This is a simulation-based replication of the real data analysis in Figure S11.

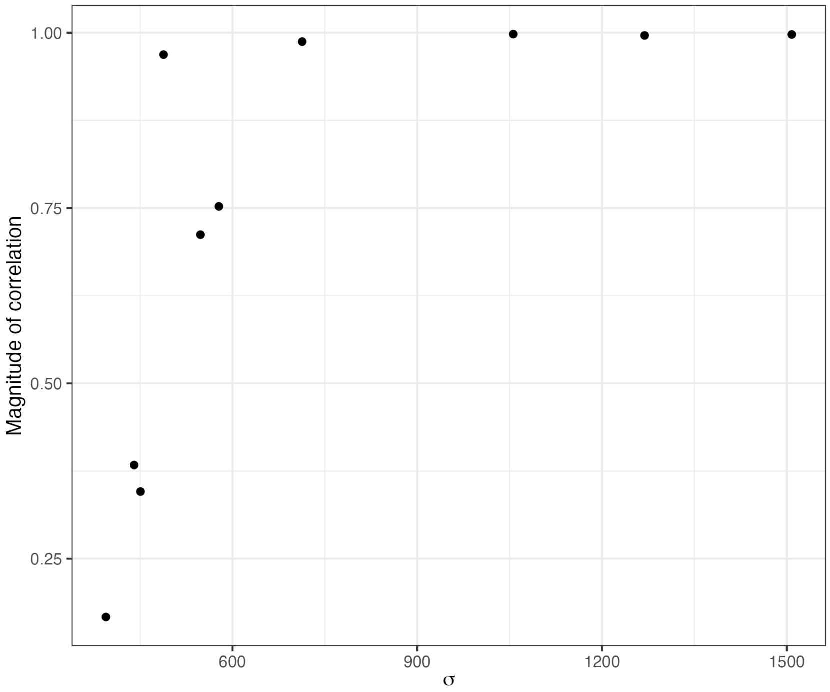

Fig. S13. Running the experiment of Figure S11 (10X immune dataset) with 100% subset size and comparing the magnitude of the correlation between scGBM-full and scGBM-proj as a function of σ_m (for scGBM-full).

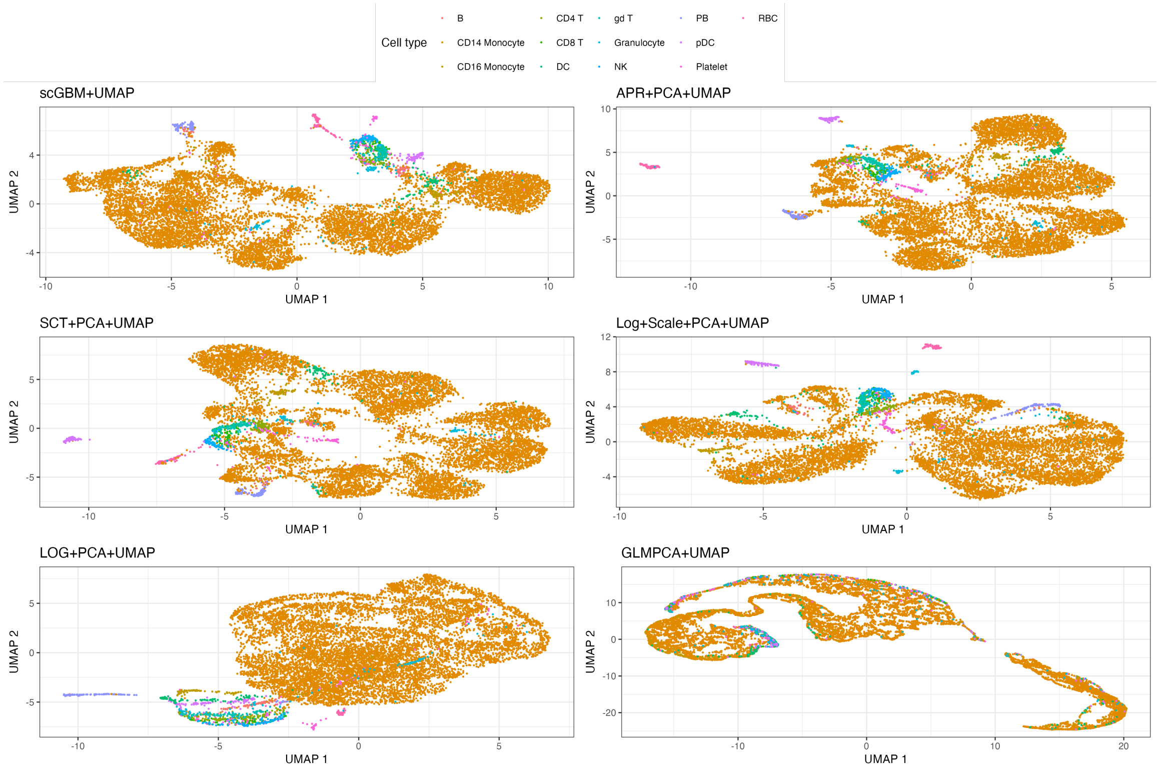

Fig. S14. UMAP embeddings on the subsampled the COVID-19 data. Details of the subsampling procedure are provided in Table S3 as well as the number of the Louvain clusters estimated by each method.

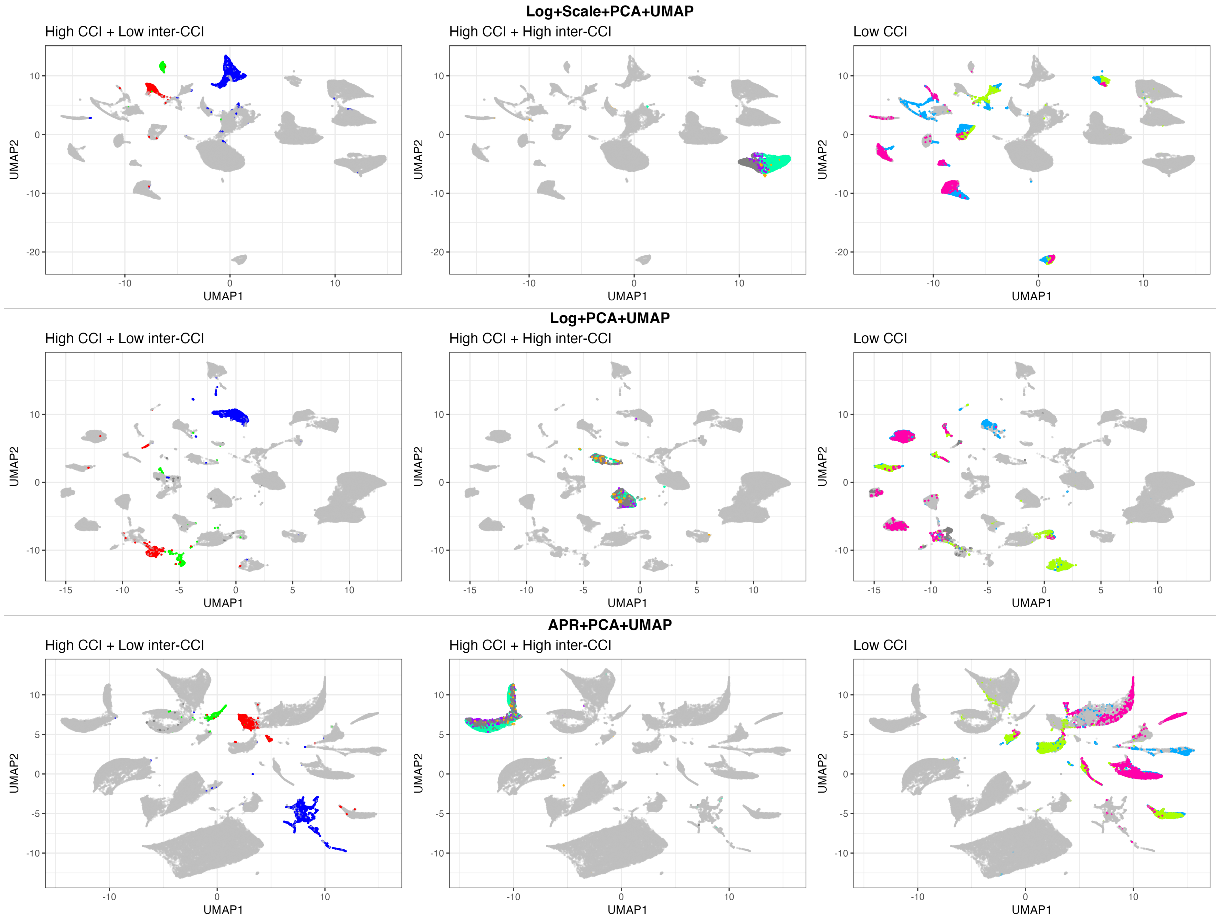

Fig. S15. For each PCA-based method, we applied UMAP and colored by three cell types with high CCI + low inter-CCI (Luminal progenitors, Myoepithelial, Plasmablasts), high CCI + high inter-CCI (Endothelial LYVE1, ACKR1, RGS5), and low CCI (Cancer Basal SC, Cycling, HER2 SC). The CCIs are computed using scGBM, because the other methods do not provide uncertainty quantification with which to compute CCIs. The colors match that of Figure 5b-c.

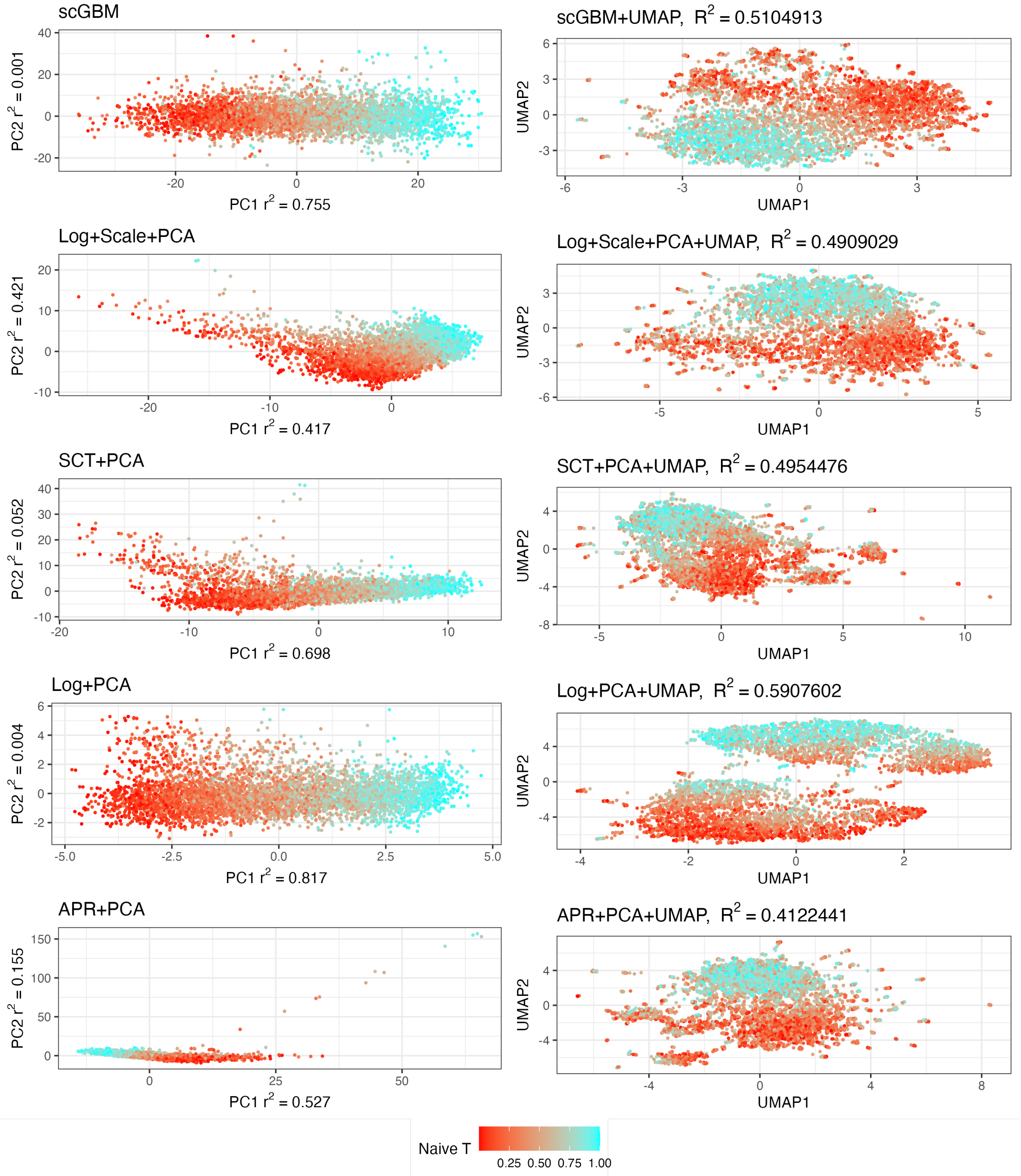

Fig. S16. Comparing methods on semi-simulated data where cells are drawn to lie on a gradient between B cells and Naive T cells. The r^2 value is the squared correlation between the embedding dimension and the true mixture proportion I'/I. For the UMAP embeddings, we report the R^2 of a linear model regressing mixture proportion against the two embedding coordinates.

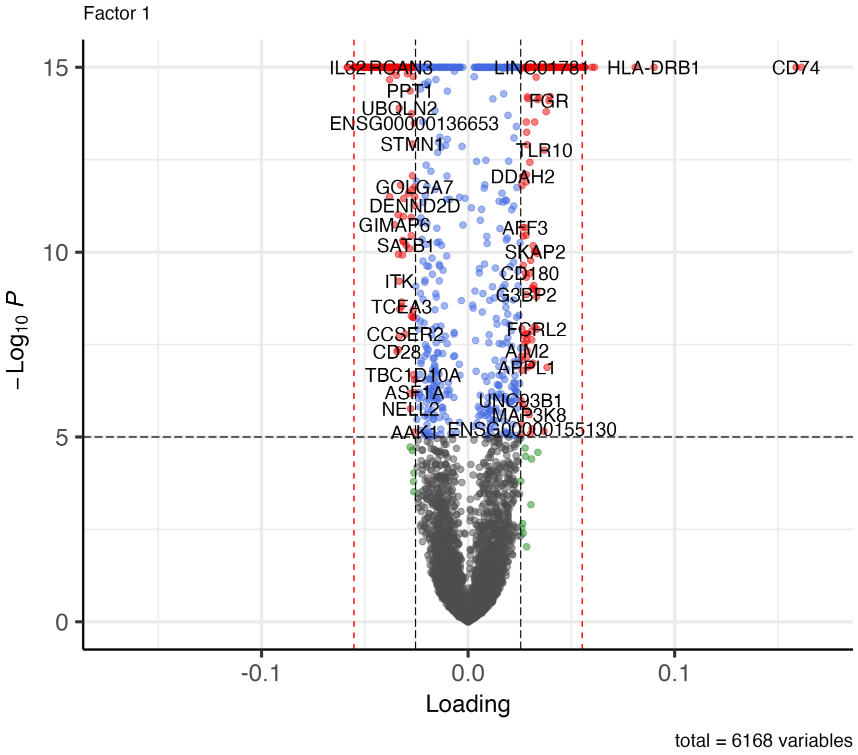

Fig. S17. A “volcano” plot of the genes driving the first scGBM factor. Specifically, the loading weight for each gene is plotted against its $-\log_{10}$ p-value. This plot was made using the *EnhancedVolcano* R package (Blighe *and others*, 2023).

References

K. Blighe, S. Rana, and M. Lewis. *EnhancedVolcano: Publication-ready volcano plots with enhanced colouring and labeling*, 2023. URL `https://bioconductor.org/packages/EnhancedVolcano`. R package version 1.18.0.

S. Correia, P. Guimarães, and T. Zylkin. Verifying the existence of maximum likelihood estimates for generalized linear models. *arXiv preprint arXiv:1903.01633*, 2019.

A. Duò, M. D. Robinson, and C. Soneson. A systematic performance evaluation of clustering methods for single-cell RNA-seq data. *F1000Research*, 7, 2018.

C. Eckart and G. Young. The approximation of one matrix by another of lower rank. *Psychometrika*, 1(3): 211–218, 1936.

C. Hafemeister and R. Satija. Normalization and variance stabilization of single-cell RNA-seq data using regularized negative binomial regression. *Genome Biology*, 20(1):1–15, 2019.

N. Halko, P.-G. Martinsson, and J. A. Tropp. Finding structure with randomness: Probabilistic algorithms for constructing approximate matrix decompositions. *SIAM Review*, 53(2):217–288, 2011.

F. Li, W. Won, E. Becker, J. Easlick, E. Tabengwa, R. Li, M. Shakhmatov, K. Honjo, P. Burrows, and R. Davis. Emerging roles for the FCRL family members in lymphocyte biology and disease. *Fc Receptors*, pages 29–50, 2014.

A. Lun and M. Morgan. *TENxBrainData: Data from the 10X 1.3 Million Brain Cell Study*, 2020. R package version 1.8.0.

J. W. Miller and S. L. Carter. Inference in generalized bilinear models. *arXiv preprint arXiv:2010.04896*, 2020.

Y. E. Nesterov. A method for solving the convex programming problem with convergence rate $O(1/k^2)$. In *Dokl. Akad. Nauk SSSR,*, volume 269, pages 543–547, 1983.

I. Razenshteyn, Z. Song, and D. P. Woodruff. Weighted low rank approximations with provable guarantees. In *Proceedings of the Forty-Eighth Annual ACM Symposium on Theory of Computing*, pages 250–263, 2016.

R. Satija, J. A. Farrell, D. Gennert, A. F. Schier, and A. Regev. Spatial reconstruction of single-cell gene expression data. *Nature Biotechnology*, 33(5):495–502, 2015.

N. Srebro and T. Jaakkola. Weighted low-rank approximations. In *Proceedings of the 20th International Conference on Machine Learning (ICML)*, pages 720–727, 2003.

E. Tuzhilina and T. Hastie. Weighted low rank matrix approximation and acceleration. *arXiv preprint arXiv:2109.11057*, 2021.

M. Uhlén, L. Fagerberg, B. M. Hallström, C. Lindskog, P. Oksvold, A. Mardinoglu, Å. Sivertsson, C. Kampf, E. Sjöstedt, A. Asplund, et al. Tissue-based map of the human proteome. *Science*, 347(6220):1260419, 2015.

F. A. Van Eeuwijk. Multiplicative interaction in generalized linear models. *Biometrics*, pages 1017–1032, 1995.

A. J. Wilk, A. Rustagi, N. Q. Zhao, J. Roque, G. J. Martínez-Colón, J. L. McKechnie, G. T. Ivison, T. Ranganath, R. Vergara, T. Hollis, et al. A single-cell atlas of the peripheral immune response in patients with severe COVID-19. *Nature Medicine*, 26(7):1070–1076, 2020.

S. Z. Wu, G. Al-Eryani, D. L. Roden, S. Junankar, K. Harvey, A. Andersson, A. Thennavan, C. Wang, J. R. Torpy, N. Bartonicek, et al. A single-cell and spatially resolved atlas of human breast cancers. *Nature Genetics*, 53(9):1334–1347, 2021.

G. X. Zheng, J. M. Terry, P. Belgrader, P. Ryvkin, Z. W. Bent, R. Wilson, S. B. Ziraldo, T. D. Wheeler, G. P. McDermott, J. Zhu, et al. Massively parallel digital transcriptional profiling of single cells. *Nature Communications*, 8(1):1–12, 2017.
